# QStatin, a Selective Inhibitor of Quorum Sensing in *Vibrio* Species

**DOI:** 10.1128/mBio.02262-17

**Published:** 2018-01-30

**Authors:** Byoung Sik Kim, Song Yee Jang, Ye-ji Bang, Jungwon Hwang, Youngwon Koo, Kyung Ku Jang, Dongyeol Lim, Myung Hee Kim, Sang Ho Choi

**Affiliations:** a National Research Laboratory of Molecular Microbiology and Toxicology, Department of Agricultural Biotechnology, and Center for Food Safety and Toxicology, Seoul National University, Seoul, South Korea; b Infection and Immunity Research Laboratory, Metabolic Regulation Research Center, Korea Research Institute of Bioscience and Biotechnology, Daejeon, South Korea; c Department of Chemistry, Sejong University, Seoul, South Korea; University of Washington

**Keywords:** LuxR, *Vibrio*, quorum sensing, quorum-sensing inhibitor

## Abstract

Pathogenic *Vibrio* species cause diseases in diverse marine animals reared in aquaculture. Since their pathogenesis, persistence, and survival in marine environments are regulated by quorum sensing (QS), QS interference has attracted attention as a means to control these bacteria in aquatic settings. A few QS inhibitors of *Vibrio* species have been reported, but detailed molecular mechanisms are lacking. Here, we identified a novel, potent, and selective *Vibrio* QS inhibitor, named QStatin [1-(5-bromothiophene-2-sulfonyl)-1H-pyrazole], which affects *Vibrio harveyi* LuxR homologues, the well-conserved master transcriptional regulators for QS in *Vibrio* species. Crystallographic and biochemical analyses showed that QStatin binds tightly to a putative ligand-binding pocket in SmcR, the LuxR homologue in *V. vulnificus*, and changes the flexibility of the protein, thereby altering its transcription regulatory activity. Transcriptome analysis revealed that QStatin results in SmcR dysfunction, affecting the expression of SmcR regulon required for virulence, motility/chemotaxis, and biofilm dynamics. Notably, QStatin attenuated representative QS-regulated phenotypes in various *Vibrio* species, including virulence against the brine shrimp (*Artemia franciscana*). Together, these results provide molecular insights into the mechanism of action of an effective, sustainable QS inhibitor that is less susceptible to resistance than other antimicrobial agents and useful in controlling the virulence of *Vibrio* species in aquacultures.

## IMPORTANCE

Yields of aquaculture, such as penaeid shrimp hatcheries, are greatly affected by vibriosis, a disease caused by pathogenic *Vibrio* infections. Since bacterial cell-to-cell communication, known as quorum sensing (QS), regulates pathogenesis of *Vibrio* species in marine environments, QS inhibitors have attracted attention as alternatives to conventional antibiotics in aquatic settings. Here, we used target-based high-throughput screening to identify QStatin, a potent and selective inhibitor of *V. harveyi* LuxR homologues, which are well-conserved master QS regulators in *Vibrio* species. Structural and biochemical analyses revealed that QStatin binds tightly to a putative ligand-binding pocket on SmcR, the LuxR homologue in *V. vulnificus*, and affects expression of QS-regulated genes. Remarkably, QStatin attenuated diverse QS-regulated phenotypes in various *Vibrio* species, including pathogenesis against brine shrimp, with no impact on bacterial viability. Taken together, the results suggest that QStatin may be a sustainable antivibriosis agent useful in aquacultures.

## INTRODUCTION

*Vibrio* species are metabolically versatile and abundant in diverse marine environments. As commensals or mutualistic symbionts, they commonly live in or on a wide range of marine organisms, including corals, zooplankton, crustaceans, shellfish, and fish (1–3). However, they also cause infectious diseases, especially in marine animals reared in aquaculture. For example, *Vibrio harveyi* causes luminescent vibriosis in shrimp and lobsters; *V. vulnificus*, *V. anguillarum*, and *V. alginolyticus* infect several fish species; and *V. crassostreae* infects oysters (4, 5). Therefore, control of pathogenic *Vibrio* species is critical for the aquaculture industry. Antibiotics have been extensively used for this purpose but have associated resistance problems. Consequently, more-sustainable alternatives that control bacterial virulence, without directly affecting bacterial viability, have attracted attention (6, 7).

Bacterial cell-to-cell communication (known as quorum sensing [QS]) makes individual cells enter “population mode” by synchronizing gene expression according to cell density. To monitor their population, each cell produces, secretes, and detects diffusible signaling molecules, called autoinducers (AIs) (8). One of the best-characterized QS systems is present in the squid symbiont *V. fischeri*, whose QS controls luminescence. In this bacterium, LuxI produces AIs, whereas LuxR (here referred to as *V. fischeri* LuxR [LuxR_*Vf*_]), a cytosolic transcriptional regulator protein, senses them directly (9). In addition to bioluminescence, diverse bacterial phenotypes requiring group cooperation such as sporulation, competence, biofilm formation, and resistance to bacteriophage are regulated by QS. Notably, pathogenesis-associated genes in many human pathogens causing chronic diseases, such as *Pseudomonas aeruginosa* and *Staphylococcus aureus*, are also controlled by QS (10). In pathogenic *Vibrio* species, QS regulates production of exoprotease/metalloprotease that causes severe diseases in marine animal hosts (11–16). Consistent with this, QS inhibition in *V. harveyi*, *V. campbellii*, *V. parahaemolyticus*, and *V. vulnificus* results in attenuated virulence against an aquatic model host, brine shrimp (17–19). Furthermore, QS provides grazing resistance and starvation-adaptation ability to *Vibrio* species and thus supports their persistence and survival in marine environments (20, 21). Accordingly, anti-QS strategies have been explored as a way to counteract the activity of pathogenic vibrios, as well as that of other chronic human pathogens. However, to our knowledge, the precise molecular mechanisms of *Vibrio* QS inhibitors remain unknown, which limits the application of this type of strategy in the field (18, 22, 23).

Although the “LuxI-LuxR_*Vf*_ system” described above is found in a range of Gram-negative bacteria, this system is not the rule for the QS in species of the *Vibrio* genus. In fact, other types of AI synthases and membrane-bound AI receptors are used by most *Vibrio* species for which QS systems have been characterized. In this “*Vibrio* QS system,” a signaling cascade initiated from the membrane-bound receptors culminates in expression of the master transcriptional regulator of the QS regulon (24, 25). The *Vibrio* QS master regulator characterized first was *V. harveyi* LuxR (here referred to as LuxR_*Vh*_), which also controls the bioluminescence of this bacterium (26). However, LuxR_*Vh*_ and its homologues conserved in other *Vibrio* species are distinct from LuxR_*Vf*_ in terms of structure and biochemical properties. LuxR_*Vh*_ homologues include *V. vulnificus* SmcR, *V. parahaemolyticus* OpaR, *V. anguillarum* VanT, *V. cholerae* HapR, and *V. fischeri* LitR (25). In fact, expression of LuxR_*Vf*_ in *V. fischeri* is regulated directly by LitR (27, 28), supporting the idea of the central role of LuxR_*Vh*_ homologues as QS master regulators in *Vibrio* species.

Previously, we and others determined the crystal structures of SmcR and HapR, which reveal a putative ligand-binding pocket within the dimerization domain (29, 30). Since they belong to the TetR family of transcriptional regulators whose DNA-binding activity is controlled in a ligand-dependent manner (31), we hypothesized that it might be possible to identify a small molecule that would bind to the pocket and regulate the function of LuxR_*Vh*_ homologues.

In this study, we performed high-throughput screening of 8,844 compounds and identified QStatin [1-(5-bromothiophene-2-sulfonyl)-1H-pyrazole] as a potent SmcR inhibitor in *V. vulnificus*. Structural analysis of SmcR complexed with QStatin revealed that the latter binds tightly to the putative ligand-binding pocket, thereby altering the DNA-binding properties of SmcR and leading to dysregulation of the QS regulon. QStatin showed pan-QS inhibitor activity in diverse *Vibrio* species that have LuxR_*Vh*_ homologues with high sequence conservation and attenuated their virulence in an aquatic host.

## RESULTS

### Small molecules interfering with SmcR.

**FIG 1:**
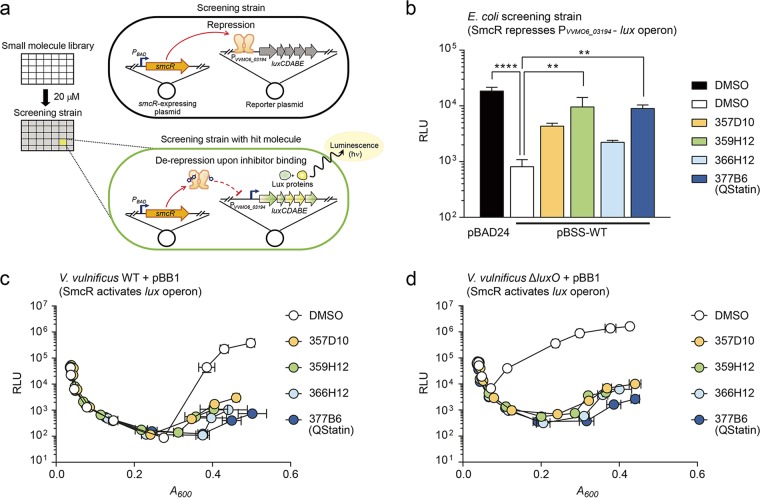
High-throughput screening for *Vibrio* QS inhibitors. (a) Strategy used to screen for a selective inhibitor of SmcR. (b to d) Verification of the hit molecules (20 μM concentration) using *E. coli* (b), *V. vulnificus* WT (c), and *V. vulnificus* Δ*luxO* (d) reporter strains. Note that the promoters of the *lux* operon in *E. coli* and *V. vulnificus* reporter strains were repressed and activated by SmcR, respectively, and that LuxO negatively regulates the expression of *smcR* in *V. vulnificus* (63). Vertical and horizontal error bars represent the standard deviations (SD) of the RLU and *A*_600_ data, respectively, from biological triplicates. Statistical significance was determined by multiple comparison after one-way analysis of variance (ANOVA) (****, *P* < 0.0001; **, *P* < 0.005).

To identify a selective inhibitor of *Vibrio* QS master regulators, we chose *V. vulnificus* SmcR as a representative target (29). A heterologous system, namely, that of *Escherichia coli*, was used to rule out the presence of false-positive molecules that inhibit QS signaling components other than SmcR. Accordingly, we cotransformed *E. coli* with the pBSS wild type (pBSS-WT) (carrying the arabinose-inducible *smcR*) and with pBS0918 (carrying the promoterless *lux* operon fused to the SmcR-repressible promoter P_*VVMO6_03194*_). This screening strain remains nonluminescent unless a potential hit molecule inhibits either the function or expression of SmcR (Fig. 1a). Screening of a total of 8,844 compounds (concentration, 20 μM) identified four hit molecules (see Fig. S1a in the supplemental material). When these molecules were reexamined at the same time, compounds 359H12 and 377B6 induced expression at significantly higher relative luminescence unit (RLU) levels than were seen with the dimethyl sulfoxide (DMSO)-treated negative-control strain. The RLUs for these compound-treated strains were almost equal to those for the positive-control strain containing the empty vector (pBAD24) instead of pBSS-WT (Fig. 1b).

10.1128/mBio.02262-17.1 FIG S1  Identification of inhibitors of SmcR activity using high-throughput screening of a chemical library. (a) A total of 8,844 compounds were screened using the *E. coli* screening strain, and four hit molecules showing substantial SmcR-inhibiting activity (>20%) were selected. The SmcR-inhibiting activity was calculated using the following equation: percent SmcR inhibition = 100 × (*y* − *z*)/(*y* − *x*), where *x* represents the average RLU value for the positive-control sample (no arabinose), *y* represents the average RLU value for the negative-control (DMSO-treated) sample, and *z* represents the RLU value for each hit molecule-treated sample. (b) Verification of the hit molecules using *V. vulnificus* Δ*smcR* reporter strains. Vertical and horizontal error bars represent standard deviations (SD) of the RLU and *A*_600_ values, respectively, from biological triplicates. (c) WT or Δ*smcR* mutant *V. vulnificus* cells were cultured to the early exponential phase (*A*_600_ = 0.25) in LBS, and 500 μl of culture was transferred to 24-well plates. The cells were then treated with QStatin (1 to 100 µM) or DMSO (2%), and placed in a 30°C shaking incubator for 10 h. The *A*_600_ level was measured every hour using a microplate reader. (d) QStatin affects the activity of the elastase gene promoter (P_*vvpE*_). QStatin activity was verified in *V. vulnificus* DH0602. In this strain, the *smcR* gene is disrupted and the elastase gene promoter (P_*vvpE*_), which is directly activated by SmcR, is transcriptionally fused to a β-galactosidase gene (*lacZ*). The strains harboring either an empty vector (pJH0311) or a SmcR-expressing plasmid (pBSJH − WT) were grown to the early exponential phase (*A*_600_ = 0.2) and treated with QStatin (20 μM) or DMSO (0.02%) as indicated. After incubation at 30°C for 16 h, β-galactosidase activity was measured. Error bars represent the SD of results from three experiments. Statistical significance was determined by one-way analysis of variance (ANOVA) (****, *P* < 0.0001). Download FIG S1, PDF file, 0.5 MB. Copyright © 2018 Kim et al. 2018 Kim et al. This content is distributed under the terms of the Creative Commons Attribution 4.0 International license.

The results were further validated using *V. vulnificus* reporter strains that harbor pBB1, a LuxR_*Vh*_-activated *lux* reporter plasmid. As previously reported for *V. harveyi* and *V. cholerae* (32, 33), the RLU level of the *V. vulnificus* WT (pBB1) decreased soon after inoculation into fresh LB supplemented with 2.0% (wt/vol) NaCl (LBS) but increased back to the initial level as the cells grew (Fig. 1c, DMSO). However, the increase of RLU was halted when the strain was treated with hit molecules (Fig. 1c). Notably, the increase of RLU was also halted when *V. vulnificus* Δ*luxO* (pBB1) was treated with the compounds (Fig. 1d), indicating that the SmcR protein, constitutively expressed in this particular strain, was the target of these compounds. Consistent with this, the compounds did not reduce the RLU level for the *V. vulnificus* Δ*smcR* (pBB1) strain (Fig. S1b).

### Identification of QStatin as an inhibitor of SmcR activity.

**FIG 2:**
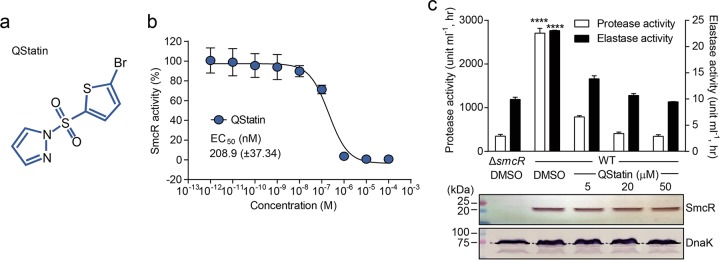
QStatin inhibits the QS master regulator SmcR. (a) The chemical structure of QStatin, 1-(5-bromothiophene-2-sulfonyl)-1H-pyrazole. (b) The EC_50_ values for QStatin were calculated from three independent experiments and expressed as means ± SD. (c) Total protease and elastase activities of *V. vulnificus* Δ*smcR* mutant and WT strains treated with DMSO or QStatin are indicated. Each activity level was measured, and the results are presented as a bar graph; data are expressed as means ± SD of results from three independent experiments. The amount of SmcR and DnaK (control) in each sample was determined by immunoblotting; one representative image is shown. Statistical significance was determined by one-way ANOVA (****, *P* < 0.0001).

Because 377B6 [1-(5-bromothiophene-2-sulfonyl)-1H-pyrazole] showed the strongest inhibition of SmcR activity without significant growth attenuation of *V. vulnificus* (Fig. S1c), it was selected as a specific SmcR inhibitor and named “QStatin” to designate its function (Fig. 2a). SmcR activity was assessed by measuring the RLU level for *V. vulnificus* WT (pBB1) in the presence of different concentrations of QStatin, which revealed that the half-maximal effective concentration (EC_50_) of QStatin was 208.9 nM (Fig. 2b).

We furthermore examined the effect of QStatin on the expression of known SmcR-activated virulence factors in *V. vulnificus*, (e.g., protease and elastase) (13, 34). As shown in Fig. 2c, the activities of both virulence factors were reduced by QStatin in a dose-dependent manner. When the β-galactosidase gene was transcriptionally fused downstream of the elastase gene promoter, P_*vvpE*_, β-galactosidase activity was also reduced by the presence of QStatin (Fig. S1d), indicating that the reduction in elastase activity by QStatin was due to its lower expression caused by SmcR dysfunction rather than to impairment of elastase catalytic activity itself. Most importantly, despite significant attenuation of SmcR function, QStatin had no effect on the cellular levels of SmcR (Fig. 2c), indicating that the compound did not affect the expression or stability of SmcR. Taken together, these results suggest that QStatin is a selective and potent inhibitor of SmcR activity.

### Molecular interaction between SmcR and QStatin.

**FIG 3:**
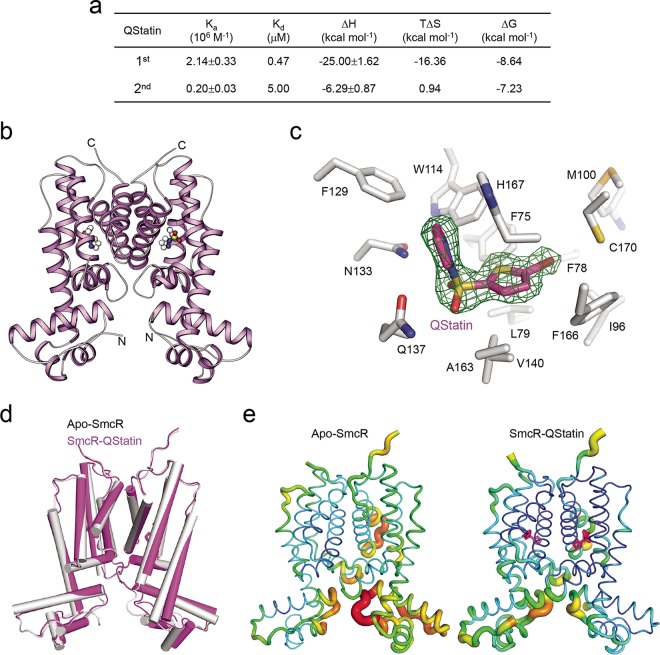
QStatin binds to the putative ligand-binding pocket of SmcR. (a) ITC analysis showing that QStatin binds directly to SmcR with high affinity. Data are representative of two experiments with similar results. *K*_a_, absorption rate constant. (b) Structure of the SmcR-QStatin complex. QStatin is represented by a ball-and-stick model. (c) Electron density difference map showing QStatin bound within the putative ligand-binding pocket of SmcR. The *F*_o_-*F*_c_ map was calculated before the inclusion of QStatin and is contoured at 3.0 σ. The SmcR residues involved in the interaction with QStatin are represented by white sticks. (d) Superimposition of the apo-SmcR and SmcR-QStatin complexes. (e) QStatin reduces the flexibility of SmcR. The structures of apo-SmcR and SmcR-QStatin were compared according to their B-factor values. High and low B-factors are represented by dark and light colors, respectively. QStatin is represented by magenta sticks.

Next, we analyzed the interaction between SmcR and QStatin by performing isothermal titration calorimetry (ITC) experiments. Exothermal reactions were observed (Fig. S2a to c) and a best fit was achieved using a two-sequential binding model. Although two identical putative ligand-binding pockets are present in the SmcR dimer (29), the sequential binding model revealed a significant difference in free energy changes; the first binding (*K*_*d*_ [dissociation constant] = 0.47 μM) was about 10-fold tighter than the second binding (*K*_*d*_ = 5.00 μM) (Fig. 3a).

10.1128/mBio.02262-17.2 FIG S2  ITC analysis of the interaction between SmcR and QStatin and verification of the biological relevance of the structural data. (a) The SmcR interaction with QStatin was examined as described in Materials and Methods. (b and c) The interactions between SmcR and buffer (b) and between buffer and QStatin (c) were also examined as control reactions. Raw data are displayed in the upper panels, and the integration plots are displayed in the lower panels. Data are representative of two experiments with similar results. (d) The H167A substitution mutation renders SmcR unaffected by QStatin. An *E. coli* screening strain expressing either WT or H167A *smcR* was treated with QStatin (20 μM) or DMSO (2%) for 6 h before measuring luminescence and *A*_600_ levels. Error bars represent the SD of the RLU values from biological triplicates. The amounts of SmcR and DnaK (control) in each sample were determined by immunoblotting; one representative image is shown. Statistical significance was determined by Student’s *t* test (****, *P* < 0.0001). Download FIG S2, PDF file, 0.5 MB. Copyright © 2018 Kim et al. 2018 Kim et al. This content is distributed under the terms of the Creative Commons Attribution 4.0 International license.

To gain further insight into the interaction between SmcR and QStatin, we determined the crystal structure of the SmcR-QStatin complex. Consistent with the results of the ITC experiments, we found that two QStatin molecules bound to the putative ligand-binding pockets of the SmcR dimer (Fig. 3b). The hydrophilic sulfonyl group and pyrazole ring of QStatin were orientated toward the pocket entrance, which was predominantly surrounded by polar residues (Fig. 3c). Specifically, the oxygen atom of the sulfonyl group and a nitrogen atom in the pyrazole ring of QStatin formed hydrogen bonds with the side chains of Asn133 and Gln137 residues, respectively. The pyrazole ring also engaged in hydrophobic interactions with the Trp114 and Phe129 residues, as well as in π-π stacking interactions with the imidazole ring of His167 (Fig. 3c). On the other hand, the bromothiophene ring of QStatin was located within the inner part of the hydrophobic ligand-binding pocket formed by Phe75, Phe78, Leu79, Ile96, Met100, Val140, Ala163, Phe166, and Cys170 (Fig. 3c). To verify the biological relevance of the interaction between SmcR and QStatin, we substituted the His167 residue involved in the interaction with QStatin with alanine. When we analyzed this substitution mutant SmcR using the *E. coli* screening strain, we found that the repressive activity of the mutant SmcR on the P_*VVMO6_03194*_ promoter was no longer affected by QStatin (20 μM), whereas that of the WT SmcR was (Fig. S2d). This indicates that the His167 residue is critical for the interaction with QStatin.

The overall structure of the SmcR-QStatin complex is quite similar to that of apo-SmcR (Fig. 3d) (29). However, the average B-factor (Å^2^) for QStatin-SmcR was 26.7, whereas that for apo-SmcR was 36.1 (Fig. 3e). The B-factor of a protein structure reflects the fluctuation of atoms around an average position and provides important information about protein dynamics (35); thus, it indicates the degree of thermal motion and static disorder of atoms in a protein crystal structure (36). Since a higher B-factor (reflected by a dark color) implies that an atom/residue is more flexible, the results indicate that QStatin binding led to a significant reduction in the flexibility of SmcR ligand-binding and DNA-binding domains, which comprised the most flexible glycine-rich hinge region (red) (Fig. 3e). These changes in flexibility may affect the interaction between SmcR and its target promoter DNAs in *V. vulnificus*.

### QStatin alters the interaction between SmcR and its target promoter DNAs.

**FIG 4:**
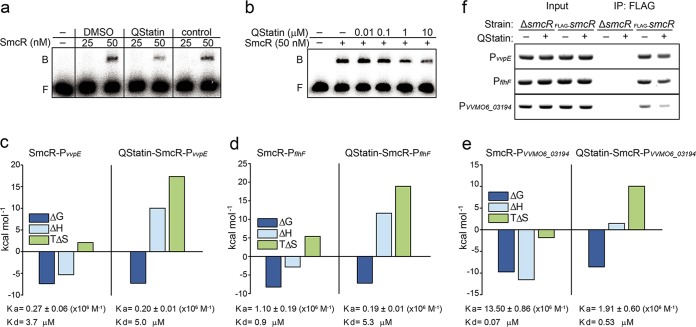
QStatin affects the interaction between SmcR and target promoter DNAs. (a) For EMSA, a 200-bp P_*vvpE*_ DNA fragment was radioactively labeled and then used as a DNA probe. Purified SmcR protein was added to the probe (15 ng) along with either QStatin (100 μM) or DMSO (2%). As a control, a random molecule (100 μm) from the library that showed no SmcR-inhibiting activity during initial screening was added instead of QStatin. B, bound DNA; F, free DNA. (b) EMSA was performed as described for panel a, except that SmcR was mixed with DMSO (2%) or increasing amounts of QStatin as indicated. (c to e) QStatin alters the interaction between SmcR and its target promoter DNAs. The promoter DNA of *vvpE* (c), *flhF* (d), or *VVMO6_03194* (e) was titrated with the apo-SmcR complex (left) or the SmcR-QStatin complex (right). The thermodynamic values calculated from the reactions are shown. Data are representative of two experiments with similar results. (f) The *in vivo* interaction between SmcR and the promoter DNAs in the presence (20 μM) or absence (0.02% DMSO) of QStatin was analyzed by ChIP. *V. vulnificus* Δ*smcR* was included as a control. A representative image from two independent experiments is shown.

To examine whether QStatin affects the DNA-binding activity of SmcR, we performed an electrophoretic mobility shift assay (EMSA) with P_*vvpE*_ DNA (13). Although SmcR binding to the DNA was reduced specifically by QStatin in a dose-dependent manner, the interaction was not completely abolished, even in the presence of large amounts (100 μM) of the molecule (Fig. 4a and b).

Thus, we used ITC experiments to examine other biochemical properties of the interactions between target promoter DNAs and either apo-SmcR or QStatin-SmcR. Consistent with the previous results, the binding affinity of apo-SmcR for the P_*vvpE*_ DNA (*K*_*d*_ = 3.7 μM) was only slightly higher than that of QStatin-SmcR (*K*_*d*_ = 5.0 μM). However, the characteristics of the interaction were significantly different; the former interaction was exothermic, whereas the latter was endothermic (Fig. 4c; see also Fig. S3a). Thus, the enthalpic (ΔH) and entropic (TΔS) components of the free binding energy of SmcR-DNA interaction were markedly changed by QStatin, indicating that QStatin affects the characteristics of the interaction between SmcR and P_*vvpE*_ DNA. Similarly, SmcR interactions with the promoter DNAs of the *flhF* (P_*flhF*_) and *VVMO6_03194* (P_*VVMO6_03194*_) genes, both of which are directly repressed by SmcR (37), were affected by QStatin. In these cases, the effect of QStatin was more noticeable, as the compound decreased the binding affinities of SmcR for the DNAs about 5-fold (for P_*flhF*_) or 8-fold (for P_*VVMO6_03194*_) (Fig. 4d and e; see also Fig. S3b and c).

10.1128/mBio.02262-17.3 FIG S3  QStatin alters the interactions between SmcR and its target promoter DNAs. Raw data and the integration plots for the reactions represented in Fig. 4c to e are shown. (a to c) Reactions were performed with P_*vvpE*_ DNA (a), P_*flhF*_ DNA (b), and P_*VVMO6_03194*_ DNA (c). Data are representative of two experiments with similar results. (d and e) Expression (d) and function and responsiveness (e) of FLAG-SmcR to QStatin were confirmed by immunoblotting and in a reporter assay. Download FIG S3, PDF file, 0.8 MB. Copyright © 2018 Kim et al. 2018 Kim et al. This content is distributed under the terms of the Creative Commons Attribution 4.0 International license.

Next, we used chromatin immunoprecipitation (ChIP) assays to assess whether changes in the DNA-binding affinity of SmcR upon exposure to QStatin reflect QStatin-mediated dysregulation of SmcR binding to its target promoter DNAs *in vivo*. To this end, a FLAG-tagged SmcR protein which was expressed and that functions and responds to QStatin in a manner identical to that of native SmcR (Fig. S3d and e) was immunoprecipitated with anti-FLAG magnetic beads from cross-linked *V. vulnificus* grown in the presence or absence of QStatin (20 μM). When the coprecipitated chromosomal DNAs were reverse cross-linked and amplified by PCR using primers specific for each promoter (P_*vvpE*_, P_*flhF*_, and P_*VVMO6_03194*_), all three promoters were amplified (even from the sample treated with QStatin); however, the intensities of the promoter bands were slightly weaker than intensities of those from the DMSO-treated sample (Fig. 4f). Thus, the results support the finding that QStatin-induced changes in the structural flexibility of SmcR have marked effects on the interaction between SmcR and its various target promoter DNAs, although mild changes in the affinity of the interaction with target DNAs do occur which may lead to SmcR dysfunction.

### QStatin globally affects the expression of the SmcR regulon *in vivo.*

**FIG 5:**
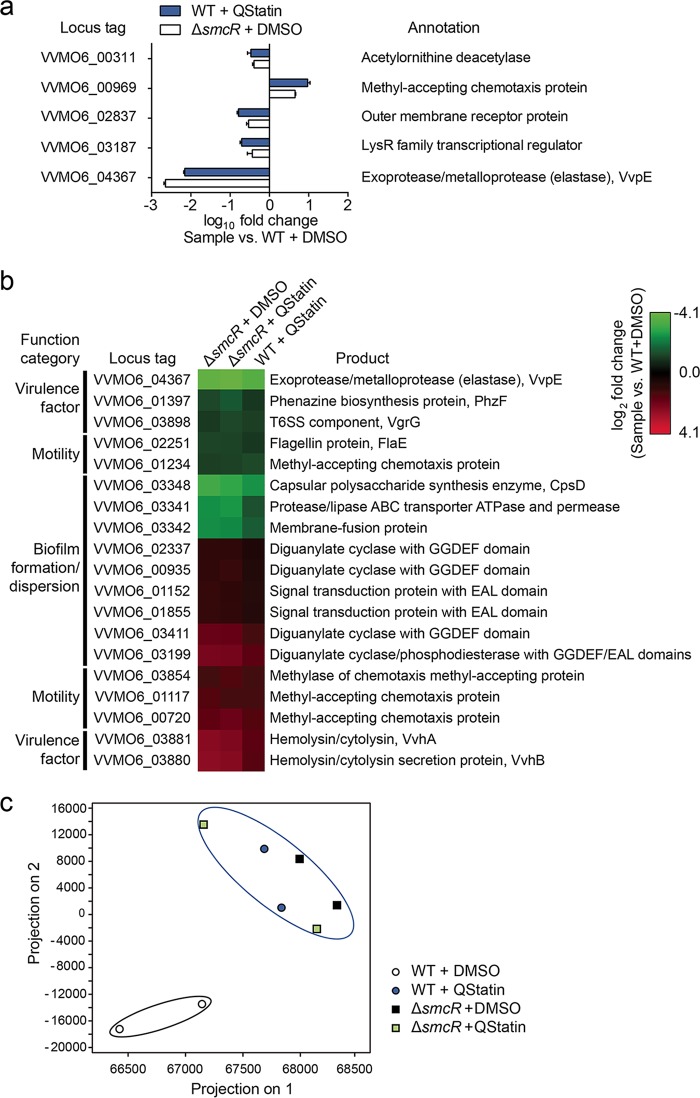
Effects of QStatin on SmcR regulon expression. (a) The fold changes in expression of each gene in either the QStatin-treated WT or DMSO-treated Δ*smcR* mutant strain are presented relative to those in the DMSO-treated WT strain. Error bars represent the SD of results from three independent experiments. (b) The fold changes of whole transcriptome expression in the DMSO-treated Δ*smcR* mutant, QStatin-treated Δ*smcR* mutant, and QStatin-treated WT biofilms relative to that in the DMSO-treated WT biofilm were examined by RNA sequencing. Among the genes differentially expressed in DMSO-treated Δ*smcR* mutant biofilm relative to DMSO-treated WT biofilm (*P* ≤ 0.05; fold change, ≥2), 19 genes potentially involved in virulence, motility, and biofilm formation/dispersion were selected. Fold changes of the expression of 19 genes in the indicated samples relative to those in the DMSO-treated WT biofilm are shown in the heat map with colors representing the log_2_ RPKM ratio. Locus tags of genes in the *V. vulnificus* MO6-24/O genome (GenBank accession numbers CP002469.1 and CP002470.1) and their gene products are shown. Please refer to Fig. S4 and Data Set S1 for expression changes in other genes. (c) Principal-component analysis of the whole-gene expression profiles of the samples. Each symbol represents the transcriptome of a single sample from two biological replicates per sample group.

We examined whether QStatin (20 μM) affects the expression of SmcR regulon *in vivo*. Both quantitative reverse transcription-PCR (qRT-PCR) and RNA sequencing analyses revealed that QStatin-treated WT *V. vulinificus* (WT+QStatin) and DMSO-treated Δ*smcR* mutant *V. vulinificus* (Δ*smcR*+DMSO) had expression profiles similar to those seen with the SmcR regulon (Fig. 5a and b; see also Fig. S4), indicating that QStatin switches SmcR to a dysfunctional state and globally affects the expression of the SmcR regulon *in vivo*. Also, QStatin does not seem to inhibit transcriptional regulators other than SmcR, because gene expression profiles in the Δ*smcR* mutant were not affected by QStatin treatment (Fig. 5b); thus, the Δ*smcR*+QStatin sample clustered with the Δ*smcR*+DMSO and WT+QStatin samples in a principal-component analysis (Fig. 5c). Taken together, these transcriptome-level *in vivo* analyses further indicate that QStatin affects the expression of the whole SmcR regulon by directly and selectively inhibiting SmcR.

10.1128/mBio.02262-17.4 FIG S4  Effects of QStatin on SmcR regulon expression. Genes differentially expressed in DMSO-treated Δ*smcR* mutant biofilm relative to those expressed in DMSO-treated WT biofilm (*P* ≤ 0.05; fold change ≥2) were identified as SmcR regulon genes. Fold changes of the expression of the genes in DMSO-treated Δ*smcR* mutant, QStatin-treated Δ*smcR* mutant, and QStatin-treated WT biofilms relative to those in DMSO-treated WT biofilm are shown in the heat map with colors representing the log_2_ RPKM ratio. Locus tags of the *V. vulnificus* MO6-24/O genome (GenBank accession numbers CP002469.1 and CP002470.1) and their gene products are shown. Download FIG S4, PDF file, 0.3 MB. Copyright © 2018 Kim et al. 2018 Kim et al. This content is distributed under the terms of the Creative Commons Attribution 4.0 International license.

10.1128/mBio.02262-17.10 DATA SET S1  Mapping statistics for the RNA sequencing reads, along with the RPKM values, fold change values, and *P* values for all genes under different conditions. Download DATA SET S1, XLSX file, 1.3 MB. Copyright © 2018 Kim et al. 2018 Kim et al. This content is distributed under the terms of the Creative Commons Attribution 4.0 International license.

### QStatin is a pan-QS inhibitor attenuating the virulence of pathogenic *Vibrio* species.

**FIG 6:**
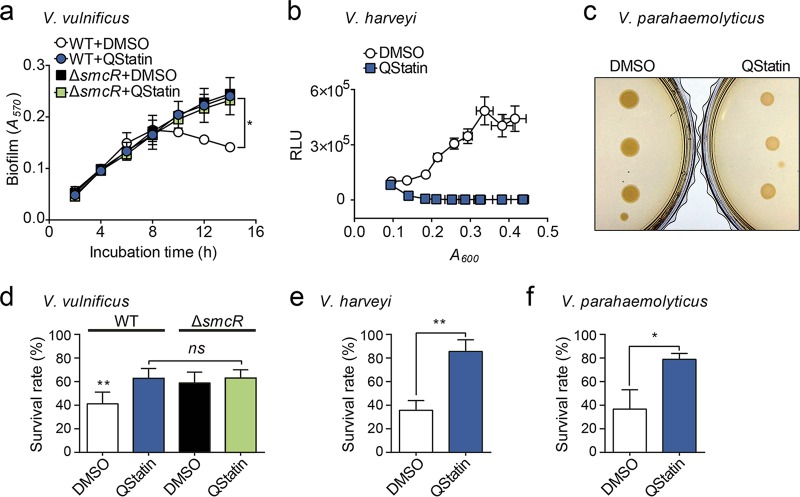
QStatin is a pan-QS inhibitor attenuating the virulence of pathogenic *Vibrio* species. (a) QStatin inhibits *V. vulnificus* biofilm dispersion. WT and Δ*smcR* mutant strains were allowed to form biofilms in the presence of QStatin (20 μM) or DMSO (0.04%) for the indicated times at 30°C. Biofilm mass was then measured by crystal violet staining. Data are expressed as means ± SD of results from two independent experiments. Statistical significance was determined by one-way ANOVA (*, *P* < 0.05). (b) QStatin inhibits *V. harveyi* bioluminescence. Early-exponential-phase *V. harveyi* cultures were transferred to microtiter plates, treated with QStatin (20 μM) or DMSO (2%), and further incubated at 30°C. Vertical and horizontal error bars represent the SD of the RLU and *A*_600_ values, respectively, from three independent experiments. (c) QStatin affects *V. parahaemolyticus* colony opacity. One microliter of an overnight *V. parahaemolyticus* culture was spotted onto LBS agar plates supplemented with QStatin (500 μM) or DMSO (2%). Three different cultures were spotted and monitored after growth at 30°C for 24 h. (d to f) Brine shrimp nauplii were challenged with *V. vulnificus* (d), *V. harveyi* (e), or *V. parahaemolyticus* (f) in the presence of QStatin (20 μM) or DMSO (0.04%). After 60 h, surviving shrimp were counted. Error bars represent the SD of the survival rates from three independent experiments. Statistical significance was determined by one-way ANOVA (d) or Student’s *t* test (e and f) (**, *P* < 0.005; *, *P* < 0.05; *ns*, not significant).

One of the known SmcR-mediated QS phenotypes in *V. vulnificus* is biofilm dispersion, which helps bacteria colonize new sites (38). Consistent with this, QStatin (20 μM) significantly impaired the biofilm dispersion of *V. vulnificus* WT, which resembled that of the DMSO-treated Δ*smcR* mutant (Fig. 6a, WT+QStatin and Δ*smcR*+DMSO). Because QStatin did not increase the mass of Δ*smcR* mutant biofilms, QStatin did not directly enhance biofilm formation but impaired SmcR-mediated biofilm dispersion (Fig. 6a, Δ*smcR*+QStatin). RNA sequencing results also showed that the expression levels of various biofilm formation/dispersion-related genes were altered similarly by QStatin treatment and the Δ*smcR* mutation (Fig. 5b).

We hypothesized that QStatin may also affect the function of other *Vibrio* LuxR_*Vh*_ homologues because the residues forming the ligand-binding pocket of SmcR were highly conserved across different LuxR_*Vh*_ homologues (Fig. 3c; see also Fig. S5a). Thus, we examined the effects of QStatin on the representative QS phenotypes of each *Vibrio* species, namely, bioluminescence of *V. harveyi* and *V. fischeri*, colony opacity of *V. parahaemolyticus*, and protease production of *V. anguillarum* (28, 39–41). All phenotypes were markedly affected by QStatin (Fig. 6b and c; see also Fig. S5b and c), indicating that this compound is a bona fide pan-QS inhibitor of *Vibrio* species.

10.1128/mBio.02262-17.5 FIG S5  Effects of QStatin on the QS-regulated phenotypes and the growth of *Vibrio* species. (a) Sequence alignments of LuxR_*Vh*_ homologues from various *Vibrio* species. The numbering shown is from SmcR. Blue triangles indicate the residues involved in the interaction with QStatin. Biological sources and GenBank accession numbers for the sequences are as follows: for SmcR, *V. vulnificus* (ADV85557.1); for LuxR_*Vh*_, *V. harveyi* (AAN86705.2); for OpaR, *V. parahaemolyticus* (NP_798895.1); for VanT, *V. anguillarum* (AAL59612.1); for HapR, *V. cholerae* (ABD24298.1); for LitR, *V. fischeri* (YP_205560.1); for LuxR_*Vm*_, *V. mediterranei* (SBO12093.1); for LuxR_*Vl*_, *V. lentus* (OCH54115.1); for LuxR_*Vs*_, *V. splendidus* (EGU44111.1); for LuxR_*Vcy*_, *V. cyclitrophicus* (OBS96501.1); for LuxR_*Vcr*_, *V. crassostreae* (CDT70654.1); and for LuxR_*Vg*_, *V. gigantis* (WP_086711963.1). Sequences alignments were assembled using Clustal Omega and visualized using ESPript 3 software. (b) An early exponential-phase *V. fischeri* culture was transferred to microtiter plates and treated with DMSO (2%) or QStatin (20 μM). Luminescence and *A*_600_ values were measured during incubation at 30°C. Vertical and horizontal error bars represent the SD of the RLU and *A*_600_ values, respectively, from three independent experiments. (c) Ten microliters of overnight-cultured *V. anguillarum* was spotted onto LBS agar supplemented with skim milk (1%) and either DMSO (0.5%) or QStatin (250 μM). Plates were photographed after 10 h of incubation at 30°C. (d) After counting the shrimp that survived (Fig. 6d to f), vibrios in the samples were enumerated. Data are expressed as means ± SD of results from three independent experiments. Statistical significance was determined by Student’s *t* test. *ns*, not significant. (e) Effect of QStatinon *Vibrio* viability in artificial sea salt solution was examined. The indicated *Vibrio* species were inoculated into a solution containing autoclaved *A. hydrophila* (10^7^ cells ml^−1^) and either 20 μM QStatin or 0.04% DMSO and were then incubated at 28°C with gentle shaking. At indicated times, samples were serially diluted and spread on LBS plates for CFU counting. Data are expressed as means ± SD of results from at least two independent experiments. (f) The indicated *Vibrio* species were cultured to the early exponential phase (*A*_600_ = 0.25) in LBS, and 1 ml of culture was transferred to 24-well plates. The cells were then treated with QStatin (20 µM) or DMSO (0.1%) and placed in a 30°C shaking incubator for 10 h. The *A*_600_ level was measured every hour using a microplate reader. Download FIG S5, PDF file, 3.7 MB. Copyright © 2018 Kim et al. 2018 Kim et al. This content is distributed under the terms of the Creative Commons Attribution 4.0 International license.

Finally, we evaluated the efficacy of QStatin against the virulence of pathogenic *Vibrio* species using their model aquatic host, *Artemia franciscana* (17–19, 42). QStatin (20 μM) markedly increased the survival rate of shrimp nauplii challenged with *V. vulnificus*, *V. harveyi*, or *V. parahaemolyticus*, without affecting the viability of bacteria (Fig. 6d to f; see also Fig. S1c and S5d to f). No such virulence attenuation was observed with the *V. vulnificus* Δ*smcR* mutant, demonstrating that virulence attenuation by QStatin is SmcR mediated (Fig. 6d). Remarkably, *V. harveyi*, a known shrimp pathogen, showed the most significant virulence attenuation; the survival rate of the nauplii increased from 35.7% ± 6.8% (DMSO) to 85.5% ± 8.1% (QStatin).

## DISCUSSION

Diverse strategies have been explored to control QS, including inhibition of AI synthesis, degradation of AI, and interference with AI detection (43). However, certain *Vibrio* species produce different kinds of AIs and sense them using specific cognate receptors (32, 44). Thus, simultaneous inhibition of all AI-specific pathways is necessary to block *Vibrio* QS. However, LuxR_*Vh*_ homologues function as master QS regulators at the center of the *Vibrio* QS pathway (25). Moreover, these homologues show high sequence similarity and may have structural similarity, making them the most attractive targets for *Vibrio* QS inhibition. QStatin inhibited SmcR activity with an EC_50_ in the range of hundreds of nanomolars and markedly affected QS in all *Vibrio* species examined. Its potent and broad-spectrum activity would be particularly important in practical settings, since multiple *Vibrio* species can cause vibriosis in aquaculture. Actually, the unit of *Vibrio* pathogenesis in naturally infected oysters is the *Vibrio* population and not the clone (45).

Some *Vibrio* species opportunistically infect human and cause acute diseases using virulence factors such as cholera toxin, toxin coregulated pili (Tcp), and hemolysin (1). Because QS represses the expression of such virulence factors (33, 46), pro-QS strategies have been proposed to treat patients (47, 48). However, this does not seem to be the case for aquatic animals, the more relevant natural hosts for *Vibrio* species (17). Indeed, virulence of *V. vulnificus* against the shrimp was considerably attenuated by QStatin (Fig. 6d) despite an increase in the expression of the hemolysin gene (Fig. 5b). This apparently conflicting result is not unprecedented, because a *V. harveyi* QS mutant also exhibits reduced virulence to brine shrimp despite expressing more type 3 secretion system (T3SS) components (17, 49). QStatin decreased the expression of many other virulence factors (Fig. 5b), including exoprotease/metalloprotease (VvpE) (19), a spike protein of T6SS apparatus (VgrG) (50), and a phenazine biosynthesis protein (PhzF) (51). Thus, these virulence factors seem to be more critical than hemolysin or T3SS in *Vibrio* pathogenesis in aquatic environments.

Furthermore, the genes governing motility (FlaE), chemotaxis (methyl-accepting chemotaxis proteins), and biofilm formation/dispersion (CabBC- and c-diGMP-regulating enzymes), which can affect both virulence and environmental adaptation of bacteria (52–54), were also significantly dysregulated by QStatin (Fig. 5b). In fact, QS has been reported to contribute to bacterial persistence and survival in the presence of grazing predators and bacteriophages in natural environments (20, 21, 55–57). Therefore, it is less likely that QStatin causes the pathogenic vibrios to bloom in the relevant environments, although the shrimp were persistently infected by vibrios under our gnotobiotic experimental conditions.

To the best of our knowledge, QStatin is the first ligand to have been shown to bind to the putative ligand-binding pocket of LuxR_*Vh*_ homologues. Furthermore, our results provide new insights into the *Vibrio* QS. First, the tight binding of QStatin to the conserved binding pocket suggests the presence of an authentic natural ligand regulating LuxR_*Vh*_ homologues. If it exists, such a ligand might have pharmacophore properties similar to those of QStatin. In this regard, it is fascinating that halogenated furanones, which bind to LuxR_*Vh*_ and affect its DNA-binding activity, are produced by the marine alga *Delisea pulchra* (58). Future examination of furanone binding to the ligand-binding pocket of LuxR_*Vh*_ homologues would reveal the relationship between *Vibrio* species and their ecologic neighborhoods at the molecular level.

**FIG 7:**
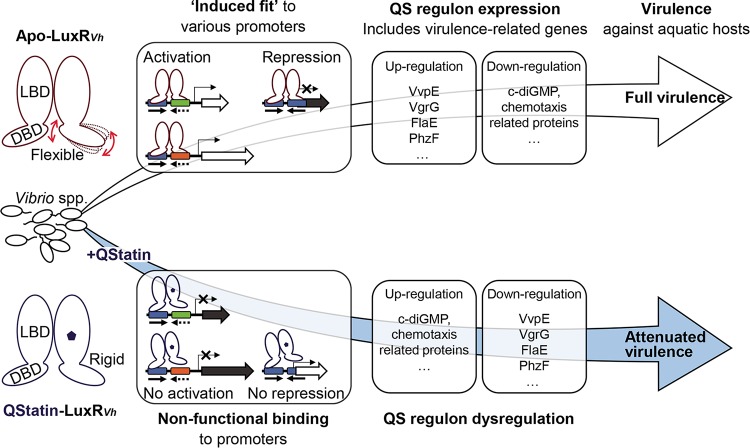
Proposed molecular mechanism underlying QStatin-induced attenuation of virulence in pathogenic *Vibrio* species. Due to its flexibility, apo-LuxR_*Vh*_ can functionally interact with various target promoter DNAs, which contain either symmetrical palindrome consensus binding sequences (represented by blue boxes with inverted arrows) or asymmetrical imperfect binding sequences (represented by blue boxes and different colored boxes) via an “induced-fit” mechanism. Virulence-related genes are differentially regulated by LuxR_*Vh*_-mediated QS, making the vibrios fully virulent against aquatic hosts. In contrast, QStatin-induced reduction of LuxR_*Vh*_ flexibility causes nonfunctional interactions with its target promoter DNAs and results in dysregulation of the LuxR_*Vh*_ regulon. These events eventually attenuate the virulence of *Vibrio* species against aquatic hosts. LBD, ligand-binding domain; DBD, DNA-binding domain.

Second, the results provide new perspectives into how LuxR_*Vh*_ homologues directly regulate many different target genes. In fact, the members of the TetR family of transcriptional regulators are known to bind to one or two promoters containing a symmetrical palindrome sequence (31). In contrast, LuxR_*Vh*_ homologues bind to hundreds of promoters harboring imperfect, asymmetrical consensus sequences in which one half is more conserved than the other half (37, 59–61). Thus, LuxR_*Vh*_ homologues are speculated to have evolved structural flexibility, allowing it to bind to less-conserved, diverse sequences (61). In the present study, we showed that QStatin reduced the structural flexibility of SmcR (Fig. 3e), altering its DNA-binding properties *in vitro* (Fig. 4a to e) and thereby dysregulating gene expression *in vivo* (Fig. 5; see also Fig. S4 in the supplemental material). Notably, QStatin affects the flexibility of the glycine-rich hinge region of apo-SmcR (Fig. 3e). Consistent with this, a previous study revealed that a natural variant of HapR with a mutation (G39D) in the glycine-rich hinge region is defective with respect to target promoter regulation (62). Thus, our results provide direct evidence that flexibility is an essential molecular feature of LuxR_*Vh*_ homologues, permitting them to function as global transcriptional regulators. If QStatin were to bind to LuxR_*Vh*_ homologues, they would become less flexible, resulting in nonfunctional binding to target promoter DNAs (Fig. 4c to f and 7). One possible explanation for this nonfunctionality is that the rigid LuxR_*Vh*_ homologues could not interact properly with other transcriptional regulators required for regulation of target promoters. Indeed, markedly rigid residues within QStatin-bound SmcR include Leu139 and Asn142, which are predicted to be essential for LuxRV_*h*_-RNA polymerase interactions (61). Since one monomer of the SmcR dimer is more flexible than the other (Fig. 3e), we propose a model in which the less flexible monomer binds to the more conserved half and the other, more flexible monomer is “induced-fitted” into the less conserved half of the consensus sequence for functional binding (Fig. 7).

In conclusion, we identified QStatin as a potent pan-QS inhibitor that selectively inhibits the activity of LuxR_*Vh*_ homologues in *Vibrio* species. Since QStatin showed a marked antivirulence effect with no direct bactericidal or bacteriostatic activity, it could be used to control vibriosis in aquacultures while avoiding the resistance associated with other antimicrobial agents. The data revealing the structure of the SmcR-QStatin complex should help us to design a more effective *Vibrio* QS inhibitor in the future. Importantly, the effect of QStatin on the persistence and survival of *Vibrio* species in real marine environments needs to be investigated, as well as any eventual mechanisms of QStatin resistance.

## MATERIALS AND METHODS

### Bacterial strains, plasmids, and culture media.

The strains and plasmids used in this study are listed in Table S1 in the supplemental material. *E. coli* and *Vibrio* strains were grown in Luria-Bertani medium (LB) at 37°C and in LB supplemented with 2.0% (wt/vol) NaCl (LBS) at 30°C, respectively, with appropriate antibiotics. The small-molecule library was generously provided by the Korea Chemical Bank (http://eng.chembank.org/), and the molecules were dissolved in DMSO. Hit molecules were either purchased from ChemDiv (San Diego, CA) or synthesized as described below. Other chemicals were purchased from Sigma-Aldrich (St. Louis, MO).

10.1128/mBio.02262-17.6 TABLE S1  Bacterial strains and plasmids used in this study. Download TABLE S1, DOCX file, 0.03 MB. Copyright © 2018 Kim et al. 2018 Kim et al. This content is distributed under the terms of the Creative Commons Attribution 4.0 International license.

### High-throughput screening.

*E. coli* DH5α was cotransformed with pBSS-WT, carrying the arabinose-inducible *smcR* gene, and with pBS0918, a reporter plasmid carrying the SmcR-repressed promoter of *VVMO6_03194* (P_*VVMO6_03194*_) (37) fused to the *lux* operon. The resulting strain was cultured to an *A*_600_ of 0.5 in fresh LB containing 0.0002% (wt/vol) l-arabinose, and then 100 μl of culture was transferred to each well of a 96-well microtiter plate (Optilux; BD Falcon, Bedford, MA) containing a 20 μM concentration of each molecule or 2% DMSO. The plates were incubated at 37°C with shaking, and luminescence and growth (*A*_600_) were measured three times at 1.5-h intervals using an Infinite M200 microplate reader (Tecan, Männedorf, Switzerland). RLU was calculated by dividing the luminescence value by the *A*_600_ value (13). Information related to the high-throughput screening is summarized in Table S2.

10.1128/mBio.02262-17.7 TABLE S2  Small-molecule screening information. Download TABLE S2, DOCX file, 0.01 MB. Copyright © 2018 Kim et al. 2018 Kim et al. This content is distributed under the terms of the Creative Commons Attribution 4.0 International license.

### Verification and determination of the EC_50_ of hit molecules.

The plasmid pBB1 carrying the LuxR_*Vh*_ homologue-activated *lux* operon (39) was conjugally transferred into the *V. vulnificus* wild-type (WT) strain, the Δ*smcR* mutant, or the Δ*luxO* mutant (63). These *V. vulnificus* reporter strains were grown overnight, diluted 1:1,000 in fresh LBS, and treated with hit molecules as described above. RLU values were calculated every hour. To determine the EC_50_, QStatin (10^−12^ to 10^−4^ M) or 2% DMSO as a control was added to the *V. vulnificus* WT reporter strain and RLU was measured after 5 h. The percentage of SmcR activity of the sample at a given concentration of QStatin was determined using the following equation: percent SmcR activity = sample RLU/control RLU × 100. The EC_50_ of QStatin (the concentration reducing the SmcR activity to 50%) was calculated from a plot of the percentages of SmcR activity versus QStatin concentrations using GraphPad Prism 6.0 (GraphPad Software, Inc., San Diego, CA).

### Determination of total protease, elastase, and β-galactosidase activities controlled by SmcR.

Total protease and elastase activities were determined as previously described (64), except that the *V. vulnificus* WT and Δ*smcR* mutant strains were treated with QStatin (5, 20, or 50 μM) or DMSO (0.02%) as described above. To confirm the reduced expression of elastase gene, *V. vulnificus* DH0602 containing SmcR-expressing plasmid pBSJH-WT (29) was treated with 20 μM QStatin for 16 h and then its β-galactosidase activity was measured as described previously (29). The amounts of cellular SmcR and DnaK were determined by immunoblotting using rat anti-SmcR polyclonal antiserum and mouse anti-*E. coli* DnaK monoclonal antibody (Enzo Lifesciences, Farmingdale, NY), respectively, with alkaline phosphatase or horseradish peroxidase (HRP)-conjugated anti-rat or anti-mouse IgG antibody, as described previously (13).

### Protein purification, crystallization, data collection, and structural analysis.

Native or selenomethionine (SeMet)-substituted SmcR was expressed and purified as described previously (29). High-quality SeMet-substituted SmcR crystals were produced under the following optimized conditions: 0.2 M Li_2_SO_4_, 7% polyethylene glycol (PEG) 3000, and 0.1 M imidazole (pH 8.0). Crystals appeared within 2 days and grew for a further 5 days. To obtain SmcR crystals complexed with QStatin, SmcR crystals were soaked for 30 min in a solution containing 2.5 mM QStatin, 0.2 M Li_2_SO_4_, 7% PEG 3000, 0.1 M imidazole (pH 8.0), and 10% glycerol. The crystals were then placed under a nitrogen gas stream (at −173°C). Diffraction data were collected at a resolution of 2.1 Å at beamline 7A (Pohang Accelerator Laboratory, Pohang, South Korea) and processed using the HKL2000 program suite (65). The structure of the SmcR-QStatin complex was solved using the molecular replacement method and the MOLREP program (66), with the SmcR structure (PDB identifier [ID]: 3KZ9) used as a template. The structure was then revised using COOT (67) and refined using REFMAC5 (68). The refinement process included the translation-liberation-screw procedure. The crystallographic data are summarized in Table S3.

10.1128/mBio.02262-17.8 TABLE S3  Data collection, phasing, and refinement statistics for the QStatin-SmcR complex. Download TABLE S3, DOCX file, 0.01 MB. Copyright © 2018 Kim et al. 2018 Kim et al. This content is distributed under the terms of the Creative Commons Attribution 4.0 International license.

### EMSA and ChIP analysis.

EMSA of SmcR binding to the *vvpE* promoter region was performed as described previously (29), except that QStatin or a random molecule from the library was added to the reaction sample. ChIP analysis was performed as described elsewhere (61), with some modifications. Briefly, the Δ*smcR* strain or a mutant strain expressing FLAG-*smcR* was grown for 16 h in the presence of either QStatin (20 μM) or DMSO (0.02%). After cross-linking with formaldehyde occurred, cells were lysed and sonicated to shear the genomic DNA. Clarified lysates were incubated for 6 h at 4°C with anti-FLAG M2 magnetic beads (Sigma-Aldrich). After washing was performed, the immunoprecipitated complexes were eluted and DNAs were reverse cross-linked. The presence of target promoter DNAs was analyzed by PCR.

### ITC analysis.

For ITC analysis of the SmcR-QStatin interaction, purified SmcR was dialyzed extensively against buffer (50 mM Tris [pH 7.0], 300 mM NaCl, 0.5% DMSO), and QStatin was diluted in the same buffer. The samples were degassed by vacuum aspiration for 15 min prior to titration at 25°C. SmcR (0.48 mM [in dimer]) in the syringe was titrated against QStatin (0.025 mM) in the reaction cell of VP-ITC (Microcal Inc., Northampton, MA). To evaluate how QStatin affects the SmcR-DNA interaction, the duplex DNAs of P_*vvpE*_, P_*flhF*_, and P_*VVMO6_03194*_ (sequences are in Table S4) were synthesized and dialyzed against the buffer. SmcR (0.42 mM [in dimer]) was incubated with QStatin (molar ratio, 1:4) prior to titration against each duplex DNA (0.02 mM). The mixture was stirred at 300 rpm, and the thermal power was recorded every 10 s. The thermograms were then analyzed using the Origin package (version 7) supplied with the instrument.

10.1128/mBio.02262-17.9 TABLE S4  Oligonucleotides used in this study. Download TABLE S4, DOCX file, 0.02 MB. Copyright © 2018 Kim et al. 2018 Kim et al. This content is distributed under the terms of the Creative Commons Attribution 4.0 International license.

### qRT-PCR.

The *V. vulnificus* strains grown to an *A*_600_ of 0.25 were treated with 20 μM QStatin or 2% DMSO and further incubated to the stationary phase (*A*_600_ = 5). Total RNA was then isolated using RNAprotect bacterial reagent and an miRNeasy minikit (Qiagen, Valencia, CA). Synthesis of cDNA and amplification of target genes were done using an iScript cDNA synthesis kit, iQ SYBR Green Supermix, and an iCycler iQ qRT-PCR system (Bio-Rad Laboratories, Hercules, CA). The sequences of the primers used are listed in Table S4. The relative expression levels of the genes were normalized to the expression of the 16S rRNA gene (internal reference), as described previously (69).

### Analysis of biofilm formation/dispersion.

Overnight-cultured *V. vulnificus* strains were diluted with *V. fischeri* minimal medium (70) containing 32.6 mM glycerol and either 20 μM QStatin or 0.04% DMSO. The diluted cultures (200 μl) were transferred to polystyrene microtiter plates (Nunc, Roskilde, Denmark) and incubated for the indicated times at 30°C to form biofilms, which were then quantitated as described previously (38).

### RNA sequencing and analysis.

Total RNA was isolated from the biofilm developed as described above, except it was incubated for 13 h in polystyrene 6-well plates (SPL, Seoul, South Korea). The RNAs were further purified by removing DNA using TURBO DNase (Ambion, Austin, TX), and mRNA was selectively enriched by depleting rRNA using a Ribo-Zero rRNA removal kit (Epicentre, Madison, WI). Then, the cDNA library was constructed using a TruSeq Stranded mRNA Sample Prep kit (Illumina, San Diego, CA). The quality of the cDNA libraries was evaluated using an Agilent 2100 Bioanalyzer and Agilent DNA 1000 reagents (Agilent Technologies, Santa Clara, CA). Strand-specific single-ended 50-nucleotide sequences were read from each cDNA library using HiSeq 2500 (Illumina). The raw sequencing reads were analyzed using CLC Genomics workbench 5.5.1 (CLC Bio, Aarhus, Denmark) and mapped onto the *V. vulnificus* MO6-24/O reference genome (GenBank accession numbers CP002469.1 and CP002470.1), allowing up to two mismatches per read. The expression level of each gene was defined using a value corresponding to the number of reads per kilobase of transcript per million mapped reads (RPKM), as described previously (71). Quantile-normalized RPKM values were then statistically analyzed by *t* tests to identify the genes that were differentially expressed (greater than 2-fold change with a *P* value of ≤0.05) from the DMSO-treated Δ*smcR* mutant cells relative to the DMSO-treated WT cells. Genes with an RPKM value of <3 were considered not to be expressed and were excluded from the analysis. Heat maps were generated by the CIMminer program (72) using the RPKM-fold change for each gene in the test samples. The mapping statistics for the sequencing reads and the RPKM values, fold change values, and *P* values for entire genes under different conditions are shown in Data Set S1 in the supplemental material. CLC Genomics workbench 5.5.1 software was used for a principal-component analysis of the whole-gene expression profiles of the samples.

### Brine shrimp challenge test.

Cysts of *A. franciscana* (INVE Aquaculture, Salt Lake City, UT) were axenically hatched and challenged as described elsewhere (17), with the following modifications. Hatched nauplii were fed with autoclaved *Aeromonas hydrophila* strain KCTC 2358 at a concentration of 10^7^ cells ml^−1^ of filtered and autoclaved artificial sea salt solution (Sigma-Aldrich) (40 g liter^−1^). The nauplii were challenged with 1 × 10^4^ CFU of *V. vulnificus* and 1 × 10^5^ CFU of *V. harveyi* or *V. parahaemolyticus* in the presence of 20 μM QStatin or 0.04% DMSO. In each experiment, at least four groups of 5 to 10 nauplii in 1 ml of the solution were transferred into each well of a 24-well plate and incubated at 28°C with gentle shaking until observation under a light microscope (Leica MZ125, Leica Microsystems, Inc., Switzerland) was performed.

### Chemical synthesis of QStatin.

Briefly, 4-bromothiophene-2-sulfonyl chloride (382 mg; 1.47 mM) and triethylamine (202 μl; 1.47 mM) were added to a solution of pyrazole (50 mg; 0.74 mM)–ethanol (10 ml). The reaction mixture was then refluxed for 6 h under a nitrogen atmosphere and cooled to room temperature. After the mixture was concentrated under reduced pressure, the crude product was extracted with methylene chloride (50 ml). The organic layer was then washed with brine, dried over Mg_2_SO_4_, and concentrated. The crude material was purified by silica gel chromatography using 10% to 30% (vol/vol) ethyl acetate in hexane as the eluent to yield QStatin as a solid (215 mg; 75% yield). The characteristics of the molecule were as follows: melting point, 112°C; ^1^H nuclear magnetic resonance (^1^H-NMR) (500 MHz, CDCl_3_) δ (ppm), 8.08 (dd, *J* = 2.8, 0.3 Hz, 1H), 7.79 (d, *J* = 1.2 Hz, 1H), 7.61 (d, *J* = 4.1 Hz, 1H), 7.09 (d, *J* = 4.1 Hz, 1H), and 6.44 (dd, *J* = 2.8, 1.6 Hz, 1H); ^13^C-NMR (126 MHz, CDCl3) δ (ppm), 145.8, 141.4, 135.1, 134.5, 131.2, 127.3, and 109.4.

### Statistical analysis.

Statistical analyses were performed as indicated in the figure legends using GraphPad Prism 6.0 software.

### Accession number(s).

The atomic coordinates and structure factors have been deposited in the Protein Data Bank (http://www.pdb.org) under PDB ID code 5X3R. All raw transcriptome data have been deposited in the NCBI BioProject database (https://www.ncbi.nlm.nih.gov/bioproject) under accession number PRJNA271541.
