# Supplementary figures and images for: QStatin, a Selective Inhibitor of Quorum Sensing in Vibrio Species
Source: mBio. 2018 Jan 30;9(1):e02262-17. doi: 10.1128/mBio.02262-17 (PMC5790914; doi:10.1128/mBio.02262-17)

a

| Chemical                                                                                                      | % SmcR-inhibition |
|---------------------------------------------------------------------------------------------------------------|-------------------|
| <b>357D10</b><br>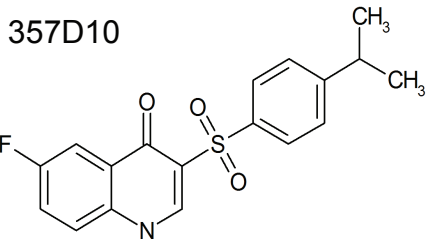            | 39.0              |
| <b>359H12</b><br>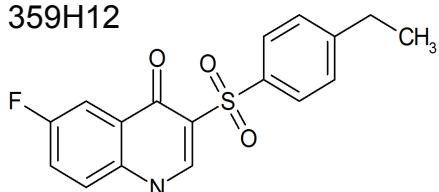            | 63.5              |
| <b>366H12</b><br>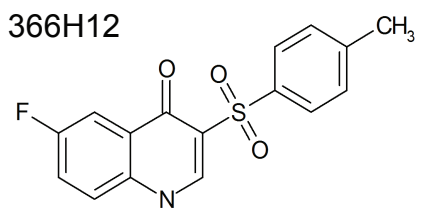           | 20.4              |
| <b>377B6 (QStatin)</b><br>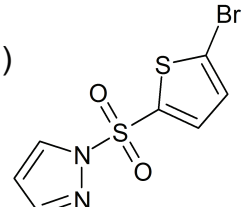 | 45.8              |

b

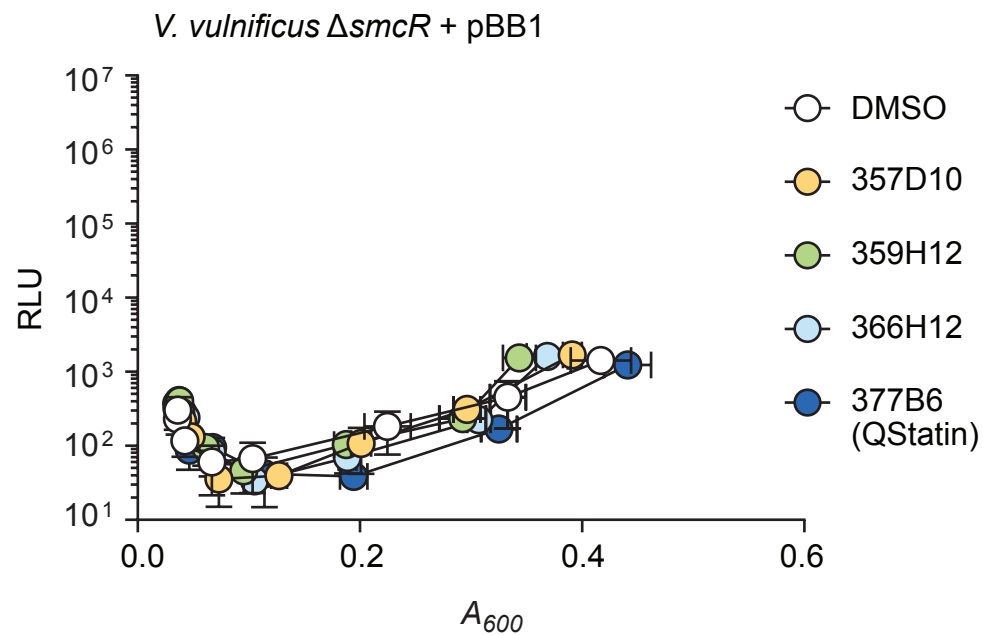

c

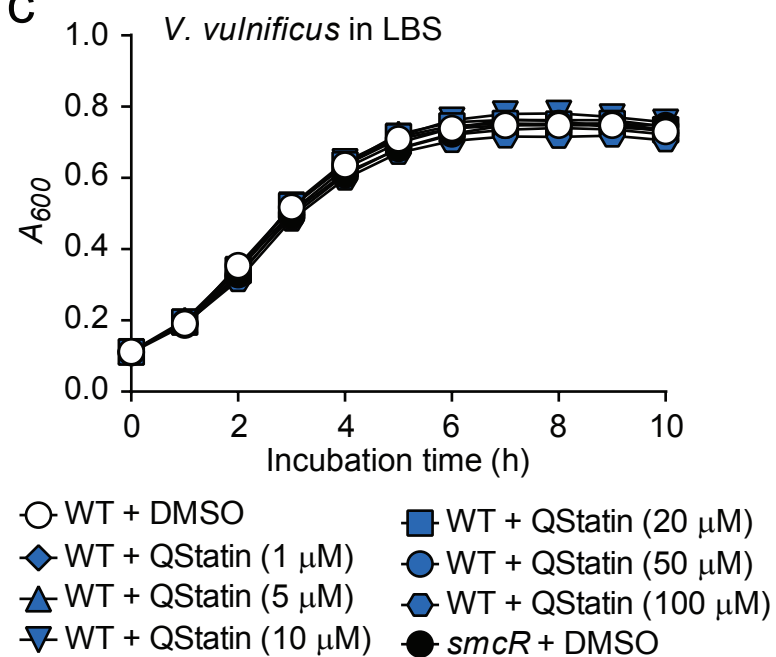

d

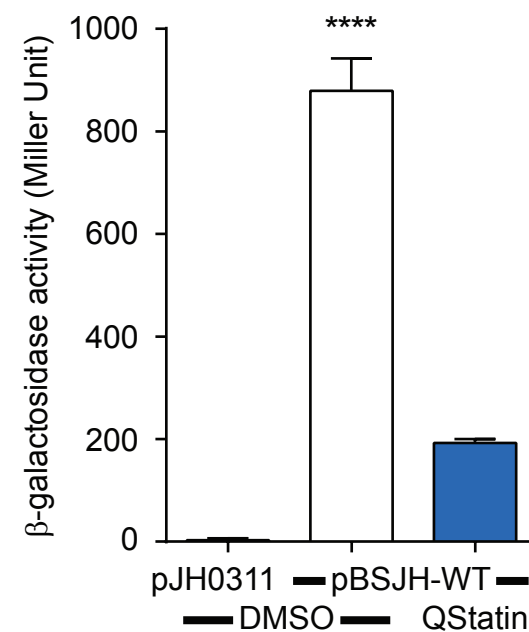Figure S1. Kim *et al.*

Supplement: FIG S1 [file mbo001183700sf1.pdf]

**a**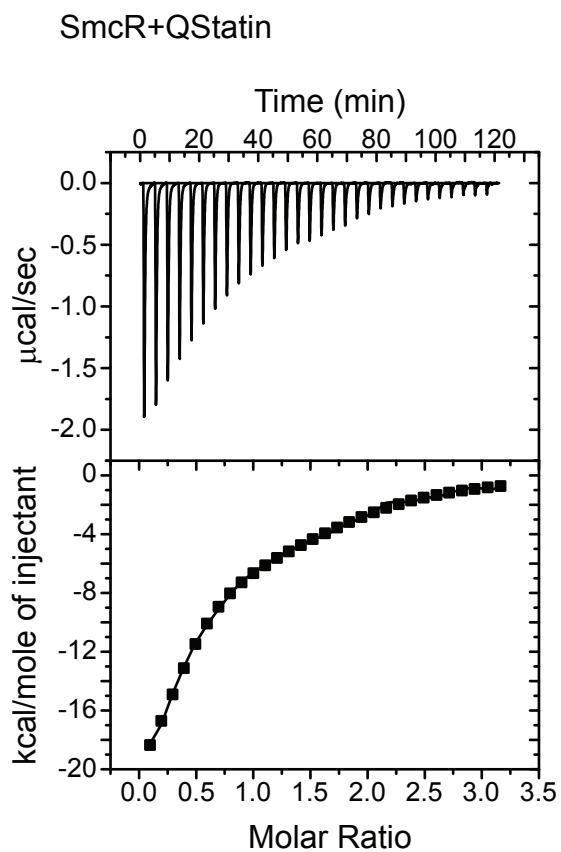**b**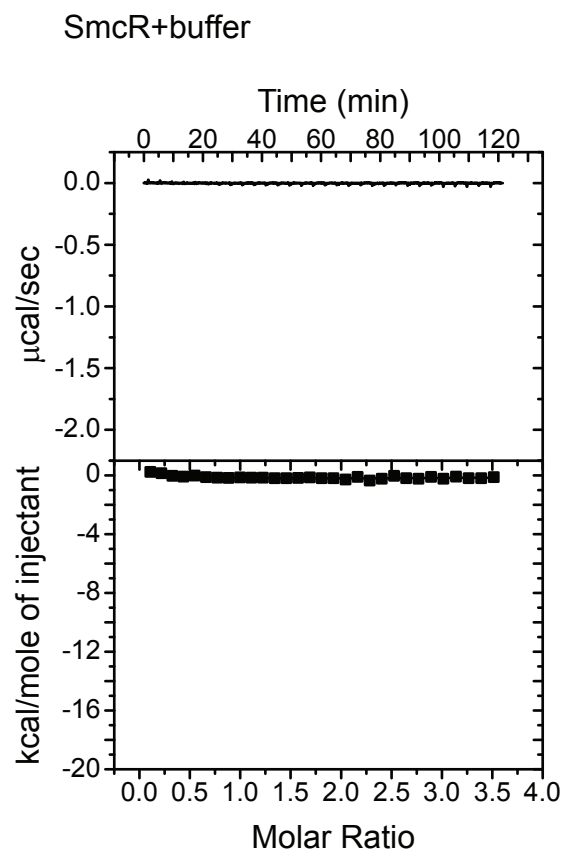**c**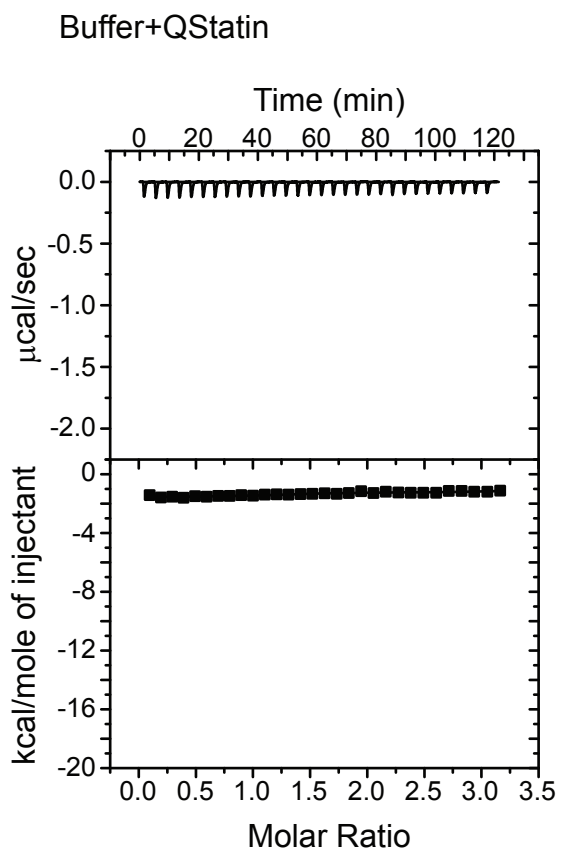**d**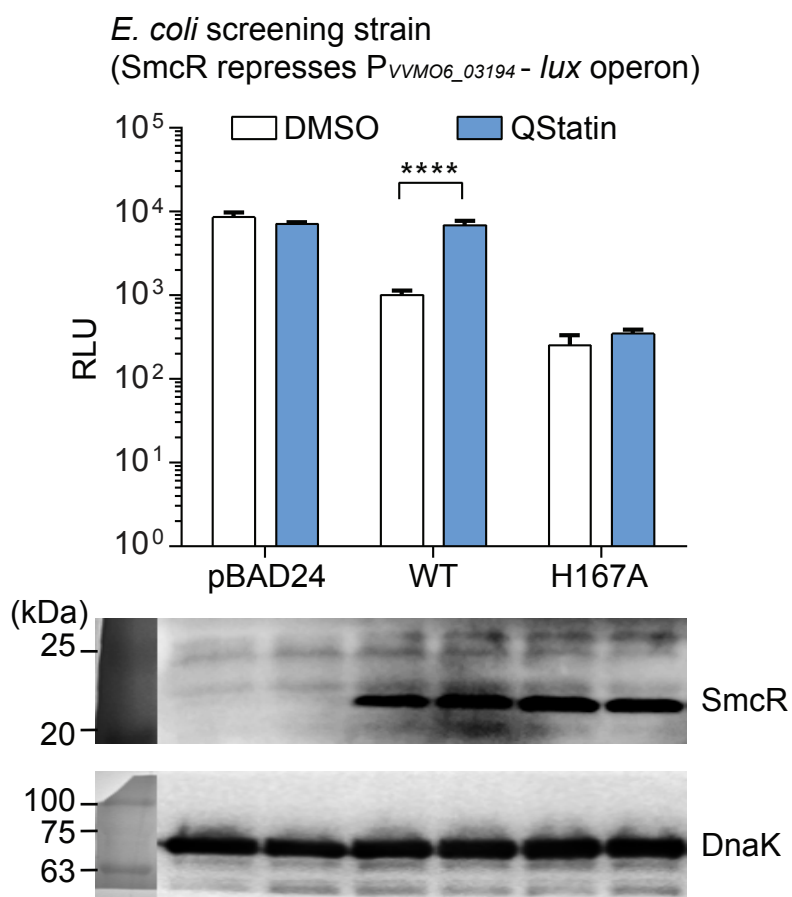Figure S2. Kim *et al.*

Supplement: FIG S2 [file mbo001183700sf2.pdf]

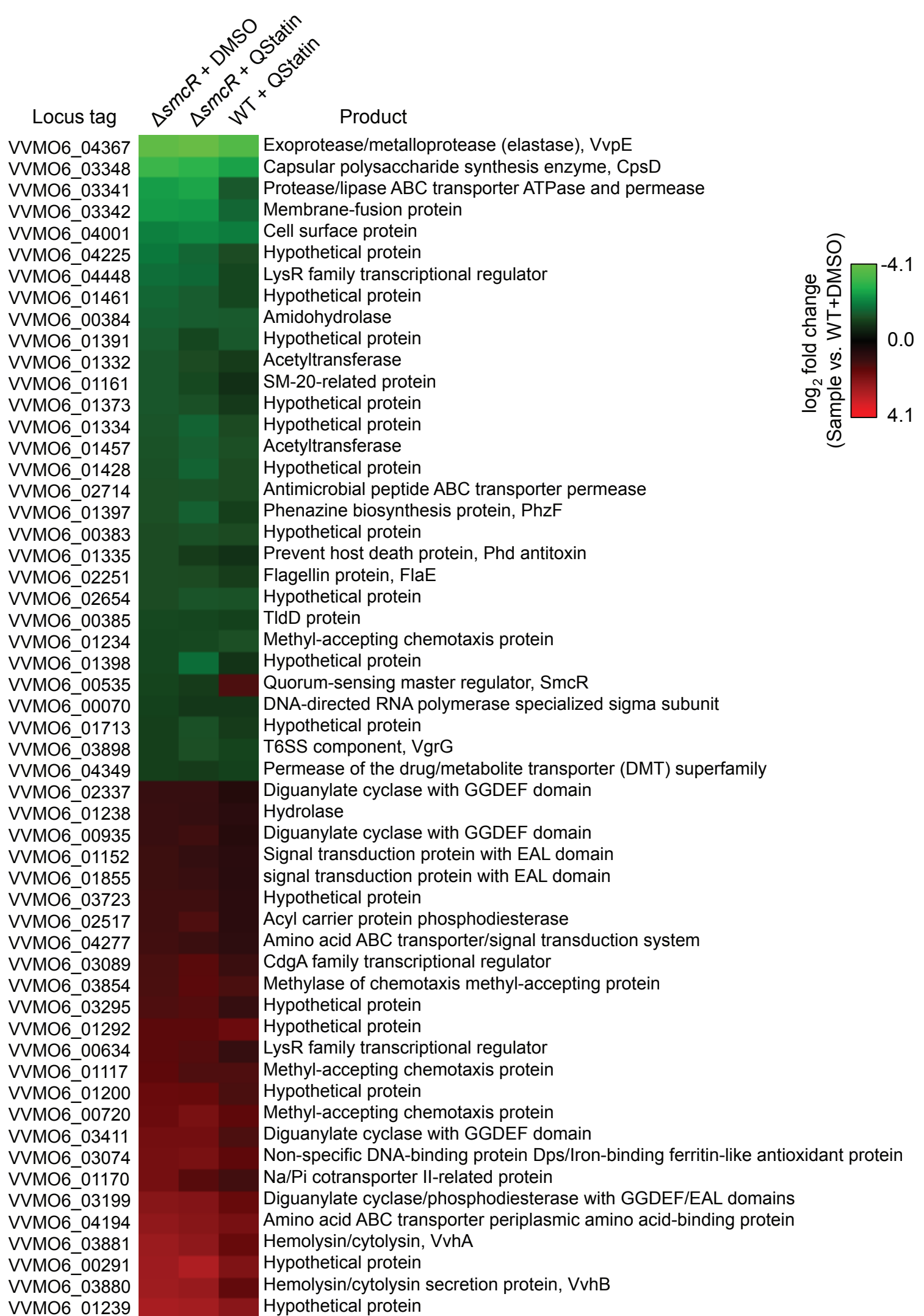

Figure S4. Kim *et al.*

Supplement: FIG S4 [file mbo001183700sf4.pdf]

a

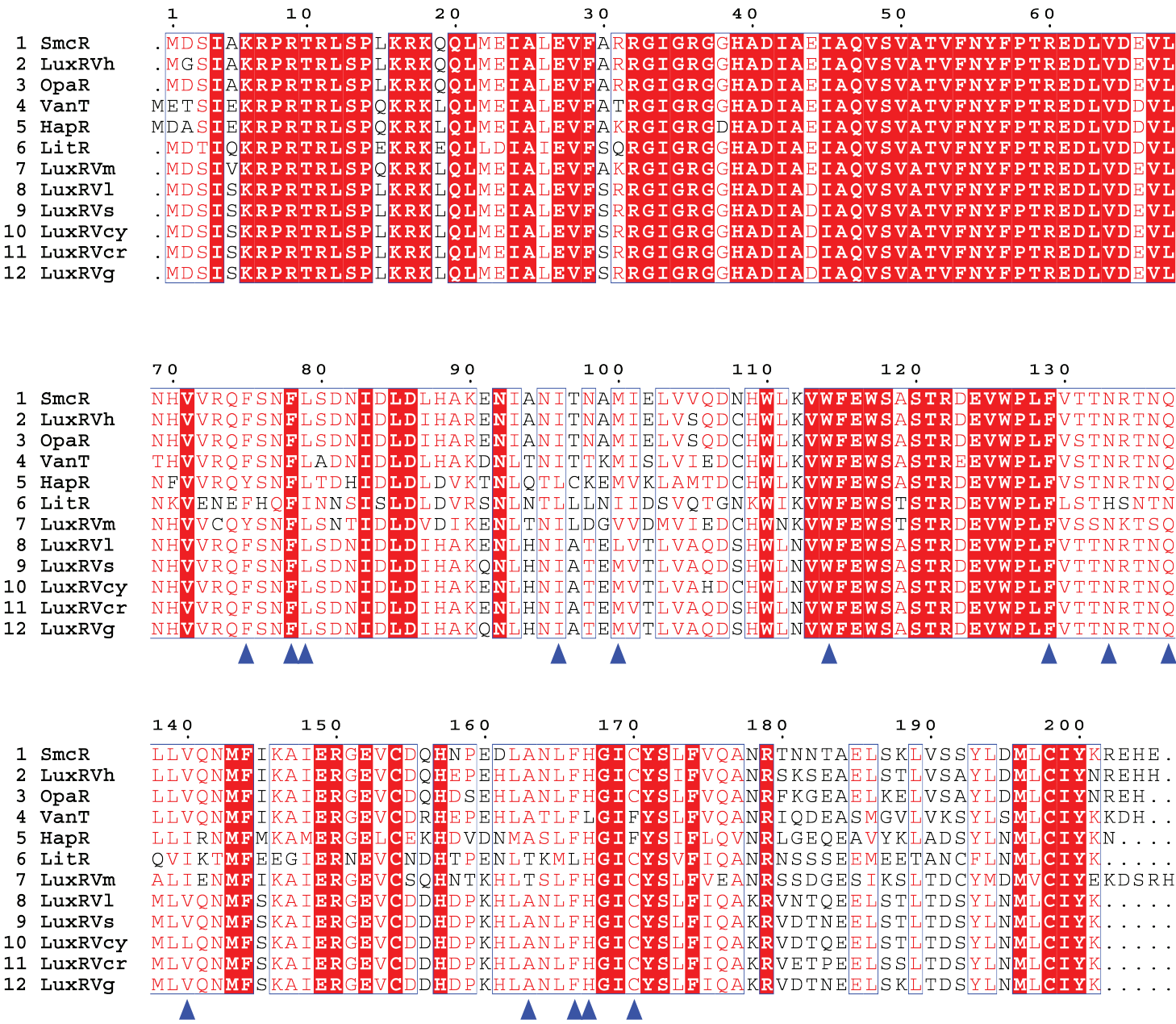

Figure S5. Kim *et al.*

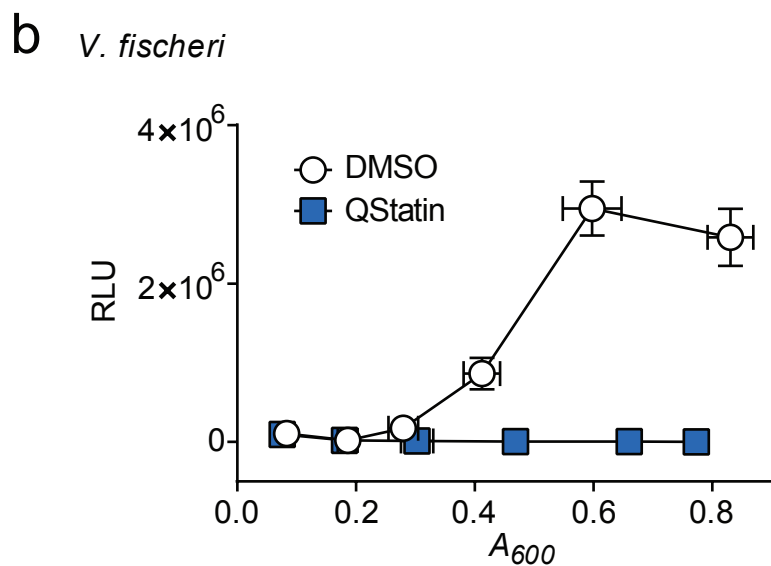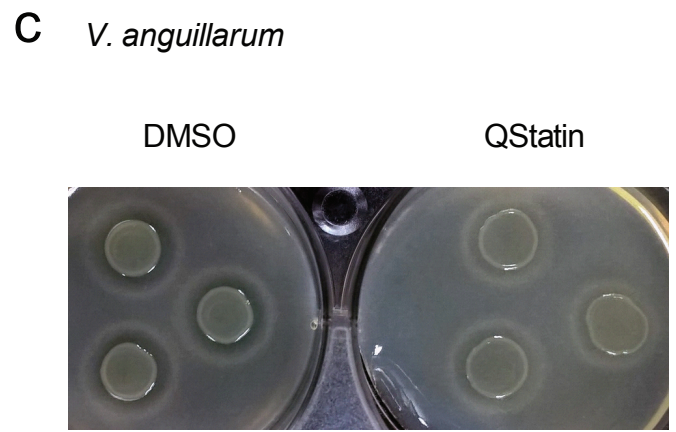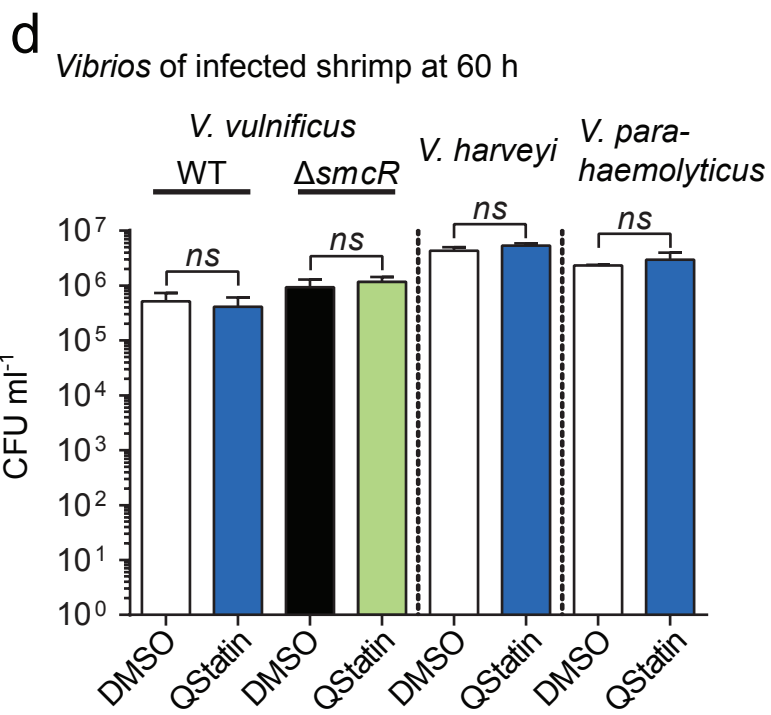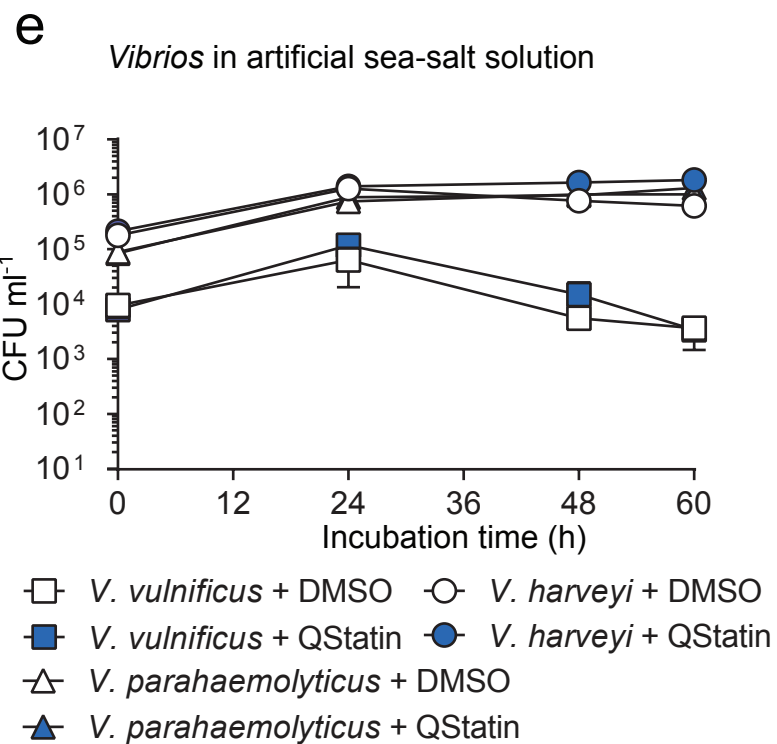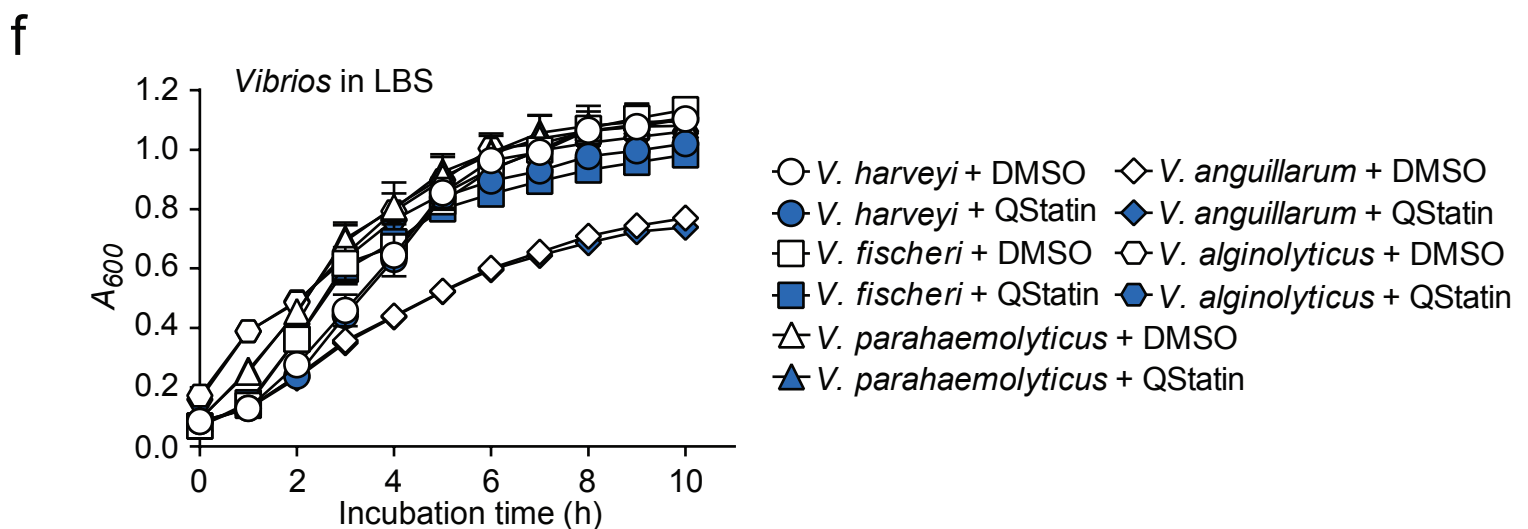

Figure S5. Kim *et al.*

Supplement: FIG S5 [file mbo001183700sf5.pdf]
