# Supplementary material for: QStatin, a Selective Inhibitor of Quorum Sensing in Vibrio Species
Source: mBio. 2018 Jan 30;9(1):e02262-17. doi: 10.1128/mBio.02262-17 (PMC5790914; doi:10.1128/mBio.02262-17)
Supplement: FIG S3 [file mbo001183700sf3.pdf]

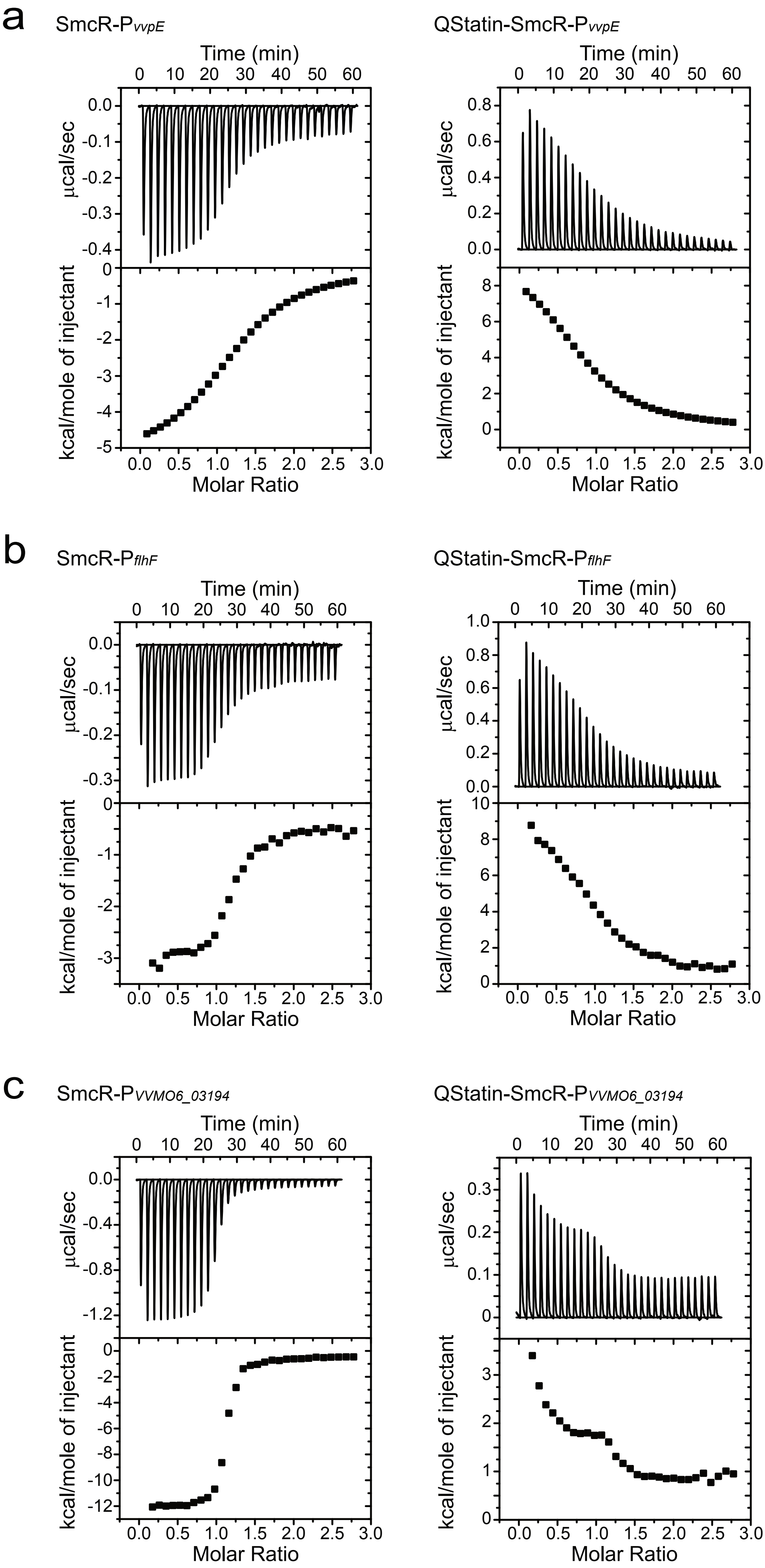

Figure S3. Kim *et al*.

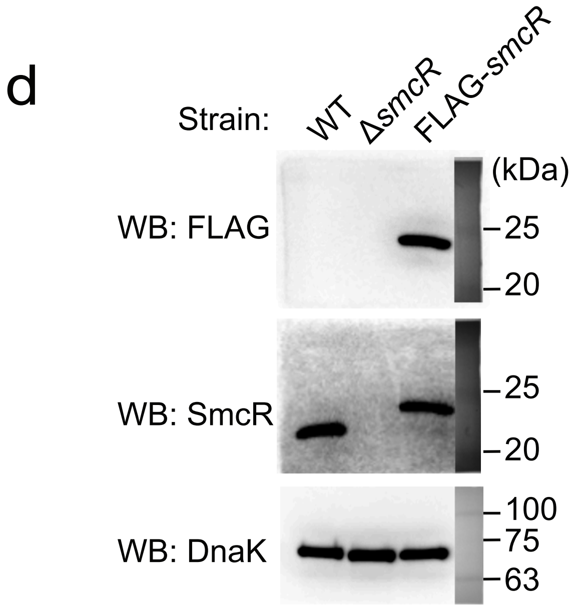

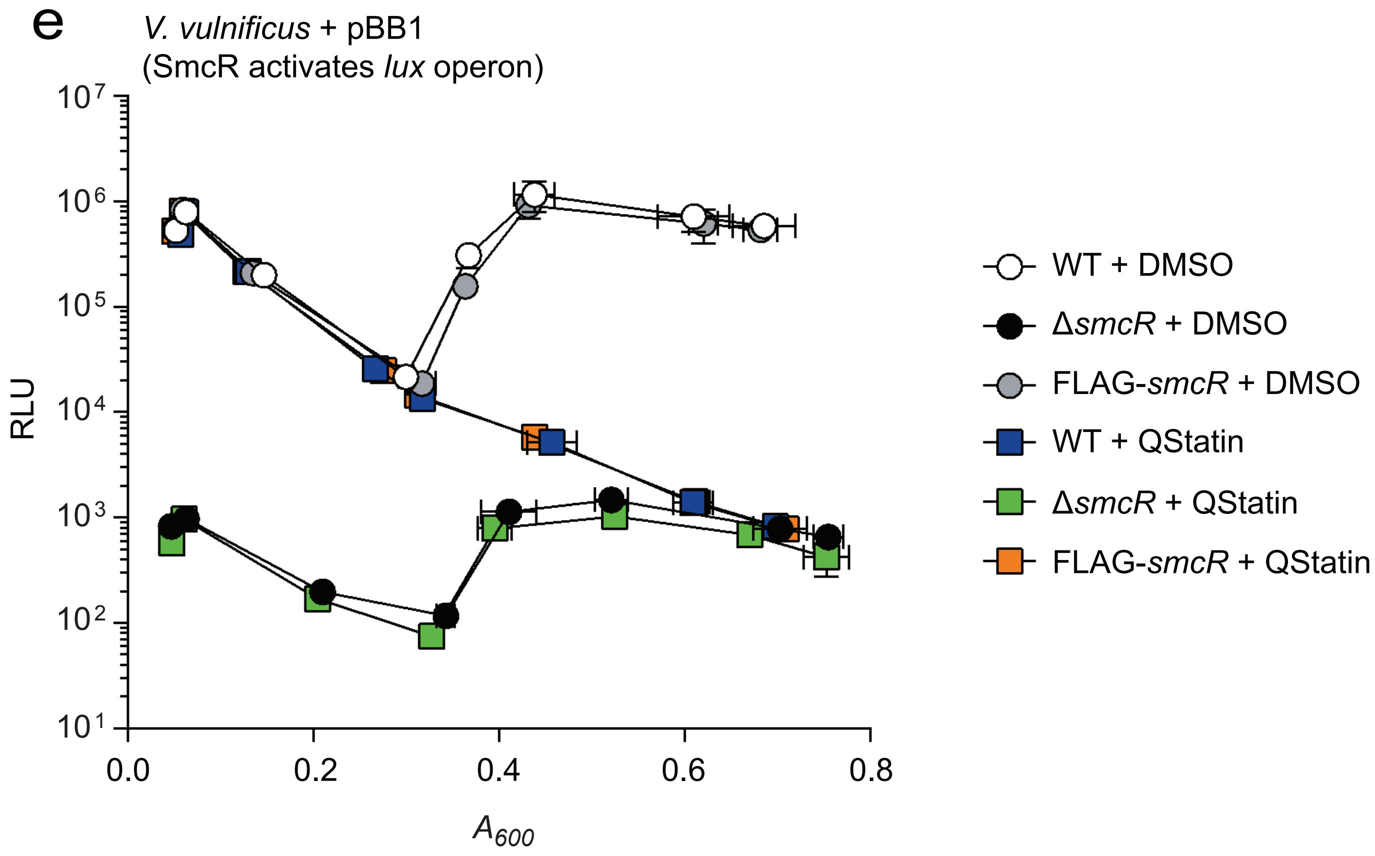

Figure S3. Kim *et al.*
