# Supplementary material for: QStatin, a Selective Inhibitor of Quorum Sensing in Vibrio Species
Source: mBio. 2018 Jan 30;9(1):e02262-17. doi: 10.1128/mBio.02262-17 (PMC5790914; doi:10.1128/mBio.02262-17)
Supplement: TABLE S1 [file mbo001183700st1.docx]

**Table S1.** Bacterial strains and plasmids used in this study

| **Strain or plasmid** | **Relevant characteristics^*^** | **Reference or source** |
| --- | --- | --- |
| **Bacterial strains** |  |  |
| *V. vulnificus* |  |  |
| MO6-24/O | Clinical isolate, virulent | Laboratory collection |
| HS03 | MO6-24/O with Δ*smcR::nptI*;Km^r^ | (1) |
| KPM201 | MO6-24/O with Δ*luxO*;Km^r^ | (2) |
| DH0602 | ATCC29307 with Δ*lacZ*, Δ*smcR*::*npt*I, *vvpE* fused to a promoterless *lacZ*; Sm^r^, Km^r^, Cm^r^ | (3) |
| BS1721 | MO6-24/O with FLAG*-smcR* | This study |
| *V. harveyi* BB120 | Wild type | (4) |
| *V. harveyi* BB886 | *luxPQ*::Tn5 | (4) |
| *V. fischeri* MJ11 | Wild type, isolated from a fish light organ | (5) |
| *V. parahaemolyticus* BB22 | Wild type, opaque variant | (6) |
| *V. anguillarum* 90-11-287 | Serotype O1, Fish pathogen | (7) |
| *V. alginolyticus* ATCC17749 | Wild type | American Type Culture Collection |
| *A. hydrophila* KCTC 2358 | Wild type | Korean Collection for Type Cultures |
|  |  |  |
| *E. coli* |  |  |
| DH5α | *supE44* Δ*lacU169* (*Φ80 lacZ* ΔM15) *hsdR17 recA1 endA1 gyrA96 thi-1 relAI* | Laboratory collection |
| SM10λpir | *thi thr leu tonA lacY supE recA*::RP4-2*-*Tc::Mu λ *pir*; Km^r^; host for π-requiring plasmids; conjugal donor | (8) |
| BL21 (DE3) | *F^―^*, *ompT*, *hsdS* (r_B_^―^, m_B_^―^), *gal* (DE3) | Laboratory collection |
|  | | |
| **Plasmids** | | |
| pBAD24 | Expression vector with the P_BAD_; Ap^r^ | (9) |
| pBSS-WT | pBAD24 with *smcR*; Ap^r^ | (3) |
| pBSS-H167A | pBAD24 with *smcR* H167A; Ap^r^ | This study |
| pBBR_lux | Broad host range vector within promoterless *luxCDABE*; Cm^r^ | (10) |
| pBS0918 | pBBR_lux harboring the promoter region of *VVMO6_03194*; Cm^r^ | This study |
| pBB1 | Broad host range cosmid pLAFR2 with *V. harveyi luxCDABE* locus; Tc^r^ | (11) |
| pJH0311 | 0.3-kb NruI fragment containing multi-cloning site of pUC19 cloned into pCOS5; Ap^r^, Cm^r^ | (3) |
| pBSJH-WT | pJH0311 with *smcR*, *rrnB* terminator; Ap^r^ | (3) |
| pFLAG-*smcR* | pDS132 with flanking regions + FLAG*-smcR*; Cm^r^ | This study |

^*^Km^r^, kanamycin-resistant; Sm^r^, streptomycin-resistant; Cm^r^, chloramphenicol-resistant; Ap^r^, ampicillin-resistant; Tc^r^, tetracycline-resistant. Antibiotics were added to the media as follows: kanamycin, 100 μg ml^-1^; streptomycin, 100 μg ml^-1^; chloramphenicol, 20 μg ml^-1^ for *E. coli* and 3 μg ml^-1^ for *V. vulnificus*; ampicillin, 100 μg ml^-1^; tetracycline, 10 μg ml^-1^ for *E. coli* and 3 μg ml^-1^ for *V. vulnificus*.

**References**

1. Kim SM, Park JH, Lee HS, Kim WB, Ryu JM, Han HJ, Choi SH. 2013. LuxR homologue SmcR is essential for *Vibrio vulnificus* pathogenesis and biofilm detachment, and its expression is induced by host cells. Infect Immun 81:3721-30.

2. Roh JB, Lee MA, Lee HJ, Kim SM, Cho Y, Kim YJ, Seok YJ, Park SJ, Lee KH. 2006. Transcriptional regulatory cascade for elastase production in *Vibrio vulnificus*: LuxO activates *luxT* expression and LuxT represses *smcR* expression. J Biol Chem 281:34775-84.

3. Kim Y, Kim BS, Park YJ, Choi WC, Hwang J, Kang BS, Oh TK, Choi SH, Kim MH. 2010. Crystal structure of SmcR, a quorum-sensing master regulator of *Vibrio vulnificus*, provides insight into its regulation of transcription. J Biol Chem 285:14020-30.

4. Henke JM, Bassler BL. 2004. Three parallel quorum-sensing systems regulate gene expression in *Vibrio harveyi*. J Bacteriol 186:6902-14.

5. Miyashiro T, Ruby EG. 2012. Shedding light on bioluminescence regulation in *Vibrio fischeri*. Mol Microbiol 84:795-806.

6. McCarter LL. 1998. OpaR, a homolog of *Vibrio harveyi* LuxR, controls opacity of *Vibrio parahaemolyticus*. J Bacteriol 180:3166-73.

7. Holmstrom K, Gram L. 2003. Elucidation of the *Vibrio anguillarum* Genetic Response to the Potential Fish Probiont Pseudomonas fluorescens AH2, Using RNA-Arbitrarily Primed PCR. J Bacteriol 185:831-842.

8. Miller VL, Mekalanos JJ. 1988. A novel suicide vector and its use in construction of insertion mutations: osmoregulation of outer membrane proteins and virulence determinants in *Vibrio cholerae* requires *toxR*. J Bacteriol 170:2575-83.

9. Guzman LM, Belin D, Carson MJ, Beckwith J. 1995. Tight regulation, modulation, and high-level expression by vectors containing the arabinose P_BAD_ promoter. J Bacteriol 177:4121-30.

10. Lenz DH, Mok KC, Lilley BN, Kulkarni RV, Wingreen NS, Bassler BL. 2004. The small RNA chaperone Hfq and multiple small RNAs control quorum sensing in *Vibrio harveyi* and *Vibrio cholerae*. Cell 118:69-82.

11. Bassler BL, Wright M, Showalter RE, Silverman MR. 1993. Intercellular signalling in *Vibrio harveyi*: sequence and function of genes regulating expression of luminescence. Mol Microbiol 9:773-86.
