# Supplementary material for: QStatin, a Selective Inhibitor of Quorum Sensing in Vibrio Species
Source: mBio. 2018 Jan 30;9(1):e02262-17. doi: 10.1128/mBio.02262-17 (PMC5790914; doi:10.1128/mBio.02262-17)
Supplement: TABLE S2 [file mbo001183700st2.docx]

**Table S2.** Small molecule screening information

| Category | Parameter | Description |
| --- | --- | --- |
| Assay | Type of assay | Whole organism (*E. coli* cells) |
|  | Target | SmcR protein |
|  | Primary measurement | Detection of bioluminescence and absorbance at 600 nm (*A_600_*) |
|  | Key reagents | L-(+)-arabinose for the induction of *smcR* gene |
|  | Assay protocol | Refer to the Materials and Methods section |
|  | Additional comments | *E. coli* cells contain the reporter plasmid with *lux* operon fused to a SmcR-repressible promoter |
| Library | Library size | 8800 used for initial screening, plus an additional 44 molecules  Arrayed in 96-well plates as single compounds at 1 mM in DMSO (total 80 compounds per plate, leaving first and second columns empty for control samples) |
|  | Library composition | Structure-representative library  (contains diverse molecules from different Pharmacophore structures (~1000); considered for drug-likeness and solubility; all compounds confirmed as >85% pure by LC-MS analysis) |
|  | Source | Korea Chemical Bank |
|  | Additional comments | For more information about the library, refer to http://eng.chembank.org/ |
| Screen | Format | 96-well plate (BD Falcon Flat Bottom Transparent/White) |
|  | Concentration(s) tested | 20 μM compound, 2% DMSO |
|  | Plate controls | Negative control: DMSO-treated *E. coli* cells plus L-(+)-arabinose; Positive control: DMSO-treated *E. coli* cells without L-(+)-arabinose |
|  | Reagent/compound dispensing system | Manual |
|  | Detection instrument and software | Infinite™ M200 microplate reader; software: Tecan i-Control ver 1.4.9.0 |
|  | Assay validation/QC | For screening validation, every plate contained positive control samples that do not express SmcR protein  SD of positive control: 29.45; SD of negative control: 7.58 |
|  | Correction factors | Luminescence was normalized against *E. coli* cell growth (*A_600_*) to obtain relative luminescence units (RLU) |
|  | Normalization | % SmcR-inhibition = 100×(y-z)/(y-x), where x is the average RLU of the positive control samples, y is the average RLU of the negative control samples, and z is the RLU of each molecule-treated sample |
|  | Additional comments | Values were measured after 4.5 h of incubation at 37°C |
| Post-screening analysis | Hit criteria | % SmcR-inhibition >20 (%) |
|  | Hit rate | 0.0452% (4 of 8,844 molecules) |
|  | Additional assay(s) | Verification of initial hits in the original (Fig. 1b) and secondary assays (Fig. 1c, d and Fig. S1b) |
|  | Confirmation of hit purity and structure | Four hit molecules were repurchased from ChemDiv. QStatin was synthesized and confirmed by ^1^H-NMR |
|  | Additional comments |  |
