# Supplementary material for: QStatin, a Selective Inhibitor of Quorum Sensing in Vibrio Species
Source: mBio. 2018 Jan 30;9(1):e02262-17. doi: 10.1128/mBio.02262-17 (PMC5790914; doi:10.1128/mBio.02262-17)
Supplement: TABLE S3 [file mbo001183700st3.docx]

**Table S3.** Data collection, phasing, and refinement statistics for the QStatin-SmcR complex

| **Data collection** |  |
| --- | --- |
| Space group | *P*2_1_2_1_2_1_ |
| Cell dimensions |  |
| *a, b, c* (Å) | 78.67, 99.21, 129.41 |
| *α, β, γ* (°) | 90, 90, 90 |
| Wavelength | 0.9795 |
| Resolution (Å) | 50-2.10 (2.14-2.10) ^†^ |
| No. total reflections | 867,700 |
| No. unique reflections | 59,830 |
| Redundancy | 14.5(14.7) |
| Completeness (%) | 99.8 (100) |
| *R*_sym_ (%)^‡^ | 8.2 (51.4) |
| I/σ(I) | 53.54 (7.78) |
| **Refinement** |  |
| Resolution (Å) | 35.0-2.10 |
| No. reflections | 56,748 |
| *R*_work_/*R*_free_^§^ | 0.19/0.25 |
| Model composition |  |
| Protein | 805 aa |
| Waters | 462 |
| Ligands | 4 QStatin, 2 sulfate |
| R.m.s. deviations |  |
| Bond lengths (Å) | 0.018 |
| Bond angles (°) | 2.044 |
| Geometry (%) |  |
| Favored region | 98.0 |
| Allowed region | 2.0 |

^†^The numbers in parentheses describe the relevant value for the highest resolution shell. ^‡^*R*_sym_ =∑ |I_i_-<I>| / ∑I where I_i_ is the intensity of the i-th observation and <I> is the mean intensity of the reflections. ^§^*R*_work_ = ∑||F_obs_| – |F_calc_|| / ∑|F_obs_|, crystallographic R factor, and *R*_free_ = ∑||F_obs_| – |F_calc_|| / ∑|F_obs_| where all reflections belong to a test set of randomly selected data.
