# Supplementary material for: QStatin, a Selective Inhibitor of Quorum Sensing in Vibrio Species
Source: mBio. 2018 Jan 30;9(1):e02262-17. doi: 10.1128/mBio.02262-17 (PMC5790914; doi:10.1128/mBio.02262-17)
Supplement: TABLE S4 [file mbo001183700st4.docx]

**Table S4.** Oligonucleotides used in this study

| **Oligonucleotide** | **Oligonucleotide sequence, 5’** **→3’^¶^** | **Location^#^** | **Use** |
| --- | --- | --- | --- |
| P*_vvpE_*_sense | ATCTTATTGATAAATCTGCGTAAAAAA | P*_vvpE_* | Duplex DNA formation for ITC |
| P*_vvpE_*_antisense | TTTTTTACGCAGATTTATCAATAAGAT | P*_vvpE_* | Duplex DNA formation for ITC |
| P*_flhF_*_sense | GTAACTGATCTATTAATTAATAACT | P*_flhF_* | Duplex DNA formation for ITC |
| P*_flhF_*_antisense | AGTTATTAATTAATAGATCAGTTAC | P*_flhF_* | Duplex DNA formation for ITC |
| P*_VVMO6_03194_*_sense | TTCTTATATTGACATATCTATAATTAACC | P*_VVMO6_03194_* | Duplex DNA formation for ITC |
| P*_VVMO6_03194_*_antisense | GGTTAATTATAGATATGTCAATATAAGAA | P*_VVMO6_03194_* | Duplex DNA formation for ITC |
| *VVMO6_03194*_B(F) | GGATCCAATGGCTTTACTTTAATCCG | P*_VVMO6_03194_* | Amplification of promoter region of *VVMO6_03194* |
| *VVMO6_03194*_B(R) | GGATCCTAACGAAGGGCTTACTGC | P*_VVMO6_03194_* | Amplification of promoter region of *VVMO6_03194* |
| VVPE021 | AGAATGGCGATTTTCATAG | P*_vvpE_* | EMSA |
| VVPE022 | GAATCCATCTCACTGCGA | P*_vvpE_* | EMSA |
| 00311_qRT_F | TCCCATCTCCCACTCTTAAC | VVMO6_00311 | qRT-PCR |
| 00311_qRT_R | CCACGCAGCAGATTATCC | VVMO6_00311 | qRT-PCR |
| 00969_qRT_F | TGTTGTAGCCGATGAAGTTC | VVMO6_00969 | qRT-PCR |
| 00969_qRT_R | ATGTATTGGATAACCTCAGCAC | VVMO6_00969 | qRT-PCR |
| 02837_qRT_F | TCGCAGAAGAGCAGCAAGTCG | VVMO6_02837 | qRT-PCR |
| 02837_qRT_R | TGGTAATGACGGTGACAGGAGAAG | VVMO6_02837 | qRT-PCR |
| 03187_qRT_F | CTCGTCTATTCAAGCCGTAA | VVMO6_03187 | qRT-PCR |
| 03187_qRT_R | TGCCATTCGCTGACAAAT | VVMO6_03187 | qRT-PCR |
| 04367_qRT_F | CAATATTGCAGCTTTAACGTCACAC | VVMO6_04367 | qRT-PCR |
| 04367_qRT_F | AACCGACCACAAGCTCTTGGG | VVMO6_04367 | qRT-PCR |
| SmcR_H167A _sense | aagatttggcgaacttgttcgccggcatttgttactcgctg | *smcR* | Site-directed mutagenesis |
| SmcR_H167A _antisense | cagcgagtaacaaatgccggcgaacaagttcgccaaatctt | *smcR* | Site-directed mutagenesis |
| P*_vvpE_*_F | AGAATGGCGATTTTCATAG | P*_vvpE_* | ChIP analysis |
| P*_vvpE_*_R | GAATCCATCTCACTGCGA | P*_vvpE_* | ChIP analysis |
| P*_flhF_*_F | AGCAGTCTTACTCACCTCCGGC | P*_flhF_* | ChIP analysis |
| P*_flhF_*_R | GACATGATGACAGCATCAACGC | P*_flhF_* | ChIP analysis |
| p*_VVMO6_03194_*_F2 | AAGAGTGTCGAATGGGCTG | P*_VVMO6_03194_* | ChIP analysis |
| p*_VVMO6_03194_*_R2 | CTAACGAAGGGCTTACTGC | P*_VVMO6_03194_* | ChIP analysis |
| FLAG-SmcR_Frag1_F | AGCTTCTTCTAGAGGTACCGCAAGATCTTCACTGCCTTGG | *smcR* upstream | FLAG*-smcR* construction |
| FLAG-SmcR_Frag1_R | TGTCGTCATCGTCTTTGTAGTCCATAGGTTGTTTCCTTACCAATG | *smcR* upstream + FLAG | FLAG*-smcR* construction |
| FLAG-SmcR_Frag2_F | CTACAAAGACGATGACGACAAGGACTCAATCGCAAAGAGACC | FLAG + *smcR* | FLAG*-smcR* construction |
| FLAG-SmcR_Frag2_R | CCCGGGAGAGCTCGATATCGAATCTTCTTAGCGAATCGTC | *smcR* downstream | FLAG*-smcR* construction |

^¶^Regions of oligonucleotides not complementary to the corresponding genes are underlined.

^#^Nucleotide hybridization site.
